# Immune- and Non-Immune-Mediated Adverse Effects of Monoclonal Antibody Therapy: A Survey of 110 Approved Antibodies

**DOI:** 10.3390/antib11010017

**Published:** 2022-02-25

**Authors:** Brian A. Baldo

**Affiliations:** 1 Kolling Institute of Medical Research, Royal North Shore Hospital of Sydney, Sydney, NSW 2065, Australia; babaldo@iinet.net.au; 2 Department of Medicine, University of Sydney, Sydney, NSW 2065, Australia

**Keywords:** approved monoclonal antibodies, monoclonal antibody adverse events, monoclonal antibody hypersensitivities, monoclonal antibody non-immune adverse events, monoclonal antibody immune adverse events, monoclonal antibody targets

## Abstract

Identification of new disease-associated biomarkers; specific targeting of such markers by monoclonal antibodies (mAbs); and application of advances in recombinant technology, including the production of humanized and fully human antibodies, has enabled many improved treatment outcomes and successful new biological treatments of some diseases previously neglected or with poor prognoses. Of the 110 mAbs preparations currently approved by the FDA and/or EMA, 46 (including 13 antibody–drug conjugates) recognizing 29 different targets are indicated for the treatment of cancers, and 66, recognizing 48 different targets, are indicated for non-cancer disorders. Despite their specific targeting with the expected accompanying reduced collateral damage for normal healthy non-involved cells, mAbs, may cause types I (anaphylaxis, urticaria), II (e.g., hemolytic anemia, possibly early-onset neutropenia), III (serum sickness, pneumonitis), and IV (Stevens–Johnson syndrome, toxic epidermal necrolysis) hypersensitivities as well as other cutaneous, pulmonary, cardiac, and liver adverse events. MAbs can provoke severe infusion reactions that resemble anaphylaxis and induce a number of systemic, potentially life-threatening syndromes with low frequency. A common feature of most of these syndromes is the release of a cascade of cytokines associated with inflammatory and immunological processes. Epidermal growth factor receptor-targeted antibodies may provoke papulopustular and mucocutaneous eruptions that are not immune-mediated.

## 1. Introduction

In the last decade, along with the continuing development of the disciplines of ge-nomics, proteomics, and bioinformatics and the application of molecular biological approaches to elucidate the functions of single genes, advances have led to insights into the complexities and multifaceted nature of diseases such as cancer, immune and inflammatory-based diseases, metabolic disorders, neurological diseases, transplantation, and some poorly understood dermatologic toxicities [1,2,3,4,5,6]. Specific, targeted approaches now employed in many monoclonal antibody (mAb), fusion protein, and cytokine therapies have been enabled by advances in recombinant DNA technology, the preparation of human recombinant antibody libraries, today’s sequencing methods, parallel proteome analyses employing techniques such as mass spectroscopy, and single B cell technologies [5,6,7]. The U.S. Food and Drug Authority (FDA) Office of Orphan Products Development and its European equivalent have provided extra stimulus for the development of therapies for “orphan diseases”, that is, diseases with less than 200,000 patients [8]. This stimulus has led to the introduction of effective approved mAb therapies for some diseases with low patient numbers previously neglected because of the lack of pathogenetic and pathophysiological insights into rare disorders where the potentially small market often precluded investigations [9].

Expanding understanding of ligand–receptor interactions; downstream signaling; and the delineation of immunological and inflammatory interplay between cells, anti-bodies, cytokines, and chemokines has contributed to the identification and selection of new disease biomarker targets. This, in turn, has created the opportunity to specifically target implicated cells, largely without inflicting collateral damage on normal healthy non-involved cells [10]. However, in addition to true hypersensitivities and infusion reactions, the expanding list of disease indications has sometimes brought with it adverse effects on the lungs, heart, liver, immune system, and skin in a variety of poorly, or partially understood, complex adverse responses [3]. A number of systemic potentially life-threatening syndromes most associated with inflammatory and immunological processes, often with cytokine involvement, also occur with low frequency during or following mAb therapy [3].

Although there are many hundreds of mAbs intended for therapeutic use at various stages of development, here we restrict examination to the 110 antibodies currently registered and approved by the U.S. Food and Drug Administration (FDA) and/or European Medicines Agency (EMA). Note, however, that some of these mAbs were first approved by other agencies while some others are already approved by other agencies but not the FDA and EMA.

Here, focus is directed to the classification of the 110 mAbs, their antibody targets, approved disease indications, and the adverse events associated with their use.

## 2. Evolution of Monoclonal Antibodies to Avoid Immunogenicity

**Figure 1:**
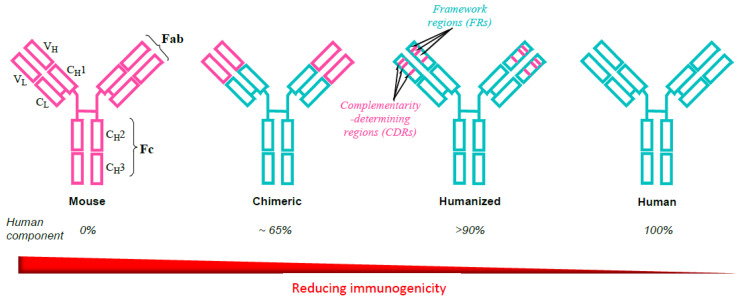
Evolution of the development of therapeutic monoclonal antibodies from murine to fully human proteins to avoid unwanted immunogenicity. The iterative process proceeded stepwise through chimeric constructs incorporating mouse immunoglobulin variable regions into constant regions of human immunoglobulins and via humanized antibodies by substituting mouse complementarity determining regions (CDRs) in place of human sequences. Fully human antibodies have been developed with the application of phage display and transgenic mice technologies. Reproduced with permission from Baldo BA. Safety of biologics therapy. Monoclonal antibodies, cytokines, fusion proteins, hormones, enzymes, coagulation proteins, vaccines, botulinum toxins. Cham, Switzerland: Springer Nature; 2016 [3].

Early realization that the murine composition of the first mAbs provoked a high incidence of adverse events including anaphylaxis and cytokine release syndrome, together with their poor pharmacokinetics, led to an ongoing iterative program to reduce, and ultimately eliminate, these undesirable features [3,11,12]. The mouse mAbs ibritumomab tiuxetan and tositumomab were soon followed by chimeric antibodies such as abciximab, cetuximab, infliximab, and others in which variable (antigen binding) regions were inserted into the constant regions of human immunoglobulins (Figure 1). Occasional serious hypersensitivities occurring after chimeric antibody infusions led to production of so-called humanized antibodies in which only approx. 5–10% of murine proteins remained after substituting mouse complementarity-determining (hypervariable) regions in place of human sequences (Figure 1). It became apparent, however, that even single amino acid changes could result in changes in antibody binding and affinity, and posttranslational glycosylation sometimes produced reductions in specificity, potency, and solubility without a reduction in immunogenicity. Development of the powerful technologies of phage display and transgenic mice finally enabled the production of fully human mAbs; however, immunogenicty can still be an occasional problem [3] due to the presence of anti-idiotype antibodies and antibodies to some mAbs (anti-glycan, anti-hinge, anti-allotype, rheumatoid factors) occurring in normal sera and sera of pretreated patients.

## 3. Monoclonal Antibody Targets and Indications

Of the 110 currently approved and registered mAbs (Table 1 and Table 2), two, alemtuzumab and denosumab, are each marketed as two separately approved products with different indications for each. Alemtuzumab, under trade names of Lemtrada^®^ and Campath^®^/MabCampath^®^ [13,14], is indicated for multiple sclerosis and B cell chronic lymphocytic leukemia, respectively, while denosumab as Prolia^®^ is indicated for bone loss and, as Xgeva^®^, for bone metastases from solid tumors and giant cell tumor of bone [15,16]. Therefore, while the total number of approved mAbs shown in Table 1 and Table 2 is 112 (66 for non-cancer and 46 for cancer therapies), alemtuzumab and denosumab each appear in both lists under different trade names.

With the steady increase in the identification and association of biomarker targets [3,17] for an expanding range of diseases, a total of 77 different targets have thus far been utilized in the preparation of the 110 currently approved mAbs with some targets complementary to more than one mAb (Table 3). In particular, there are 29 targets for the 46 different mAb cancer therapies (Table 2) and a collective of 48 targets for a diverse range of 66 mAbs for non-cancer disorders, including 27 inflammatory and/or immune disorders and 39 other diseases/applications (Table 1). For the mAbs used for non-cancer therapies, 14 different targets have been employed two or more times (Table 3). For example, TNF as target has been utilized for four mAbs—adalimumab, certolizumab pegol, golimumab, and infliximab—each used in the treatments of inflammatory diseases including rheumatoid arthritis, psoriatic arthritis, ankylosing spondylitis, plaque psoriasis, and Crohn’s disease. IL-6R serves as target for three different mAbs—sarilumab and tocilizumab, each used to treat rheumatoid arthritis, and satralizumab-mwge, indicated for a quite different condition, neuromyelitis optica spectrum disorder (Table 1 and Table 3). For the treatment of cancers, eight different targets are utilized for more than one mAb. The targets HER2, EGFR, programmed cell death protein 1 PD-1, and its ligand PD-L1 have been used as complementary targets for, respectively, five, four, three, and four different mAbs (Table 2 and Table 3).

## 4. Adverse Events to Monoclonal Antibody Therapy

Despite their target specificity, their low tendency for drug–drug interactions, and their generally better patient tolerance than small molecule drugs, mAbs are, unsurprisingly, not free of adverse effects, which may manifest as immune, non-immune, or direct cytotoxic reactions. Table 4 and Table 5 summarize adverse events associated with mAbs used for non-cancer and cancer therapies, respectively. For all mAbs, there is the possibility of injection site reactions, infusion reactions, hypersensitivity, and immunogenicity, although these effects are more likely with some mAbs than others. Many of the approved mAbs are subject to warnings for “hypersensitivity”, often without further qualification, which is generally unhelpful given the loose usage of this term and the fact that it often has a different meaning to clinicians and investigators in different branches of medicine [18,19]. Immunogenicity is always a concern even with fully human antibodies since anti-idiotype responses can occur [3,20].

Adverse events, divided into immune, that is true hypersensitivities, and non-immune, are herein considered.

### 4.1. Immune-Mediated Adverse Responses (Hypersensitivities) to Approved Monoclonal Antibodies

Collectively, patient responses to mAbs cover the full range of hypersensitivities from types I to IV (Box 1) [19] with the type I IgE-antibody-mediated hypersensitivity responses—anaphylaxis; urticaria (e.g., to ofatumumab and alemtuzumab); and, rarely, angioedema (e.g., with trastuzumab) occasionally seen. Chimeric mAbs with mouse and/or rat sequences (abciximab, basiliximab, blinatumomab, brentuximab vedotin, catumaxomab, cetuximab, dinutuximab, infliximab, obiltoxaximab, rituximab, and siltuximab) are considered to be the highest risk for type I reactions. Overall, however, reports of type I hypersensitivities are relatively rare, and perhaps less than expected, with only two FDA black box warnings issued thus far (for the humanized mAbs reslizumab and obiltoxaximab) and two FDA warning/precaution for palivizumab and brentuximab vedotin. Table 6 lists 19 mAbs with warnings for, and reports of, anaphylaxis, with 5 employed in cancer therapy (Table 5) and 14 for other disorders (Table 4). Severe infusion reactions that occur with some mAbs and which show some similar symptoms to anaphylaxis (see Section 4.2) can sometimes make distinguishing the two difficult and lead to doubts about the true incidence of anaphylaxis.

Box 1 **Hypersensitivity reactions, known and some suspected, to approved monoclonal antibodies used for therapy.**

- **TypeI hypersensitivity:** Warnings for, and reports of, anaphylaxis account for ≈18% of mAbs, 14 used for non-cancer indications and 5 for cancer indications. Reslizumab and obiltoxaximab are covered by a black box warning for anaphylaxis. Urticaria occurs more often with the non-cancer mAbs.
- Serious **infusion reactions** with signs and symptoms resembling, and sometimes confused with anaphylaxis, occur with some mAbs, for example, alemtuzumab, cetuximab, dinutuximab, ibritumomab tiuxetan, naxitamab-gqgk, panitumumab, rituximab, trastazumab, and vedolizumab. Cytokine release appears to be involved.
- There is as yet no good evidence that many cytopenias are **type II hypersensitivities,** but these may occur with, for example, abciximab, alemtuzumab for multiple sclerosis and rituximab. Autoimmune hemolytic anemia may be induced by alemtuzumab and rituximab and rituximab-induced early- and late-onset neutropenia may be immune-mediated.
- **Type III hypersensitivities**, serum sickness-like reactions, cutaneous vasculitis, and hypersensitivity pneumonitis (may be a combined type III and IV hypersensitivity) occur with, for example, infliximab, adalimumab, and alirocumab. Checkpoint inhibitors including ipilimumab, nivolumab, and avelumab (Table 5) may also induce hypersensitivity pneumonitis. Chimeric mAbs (e.g., rituximab) and the humanized mAb omalizumab may cause a serum sickness-like reaction.
- Precise mechanisms for immune-mediated colitis, hepatitis, nephritis, hypothyroidism, and endocrinopathies induced by mAbs targeted to PD-1 and PD-L1 checkpoint inhibitors are not yet established.
- **Type IV hypersensitivities**: Rare Stevens–Johnson syndrome reactions have been reported to adalimumab, brentuximab vedotin, infliximab, and rituximab; toxic epidermal necrolysis has been induced by ibritumomab tiuxetan and rituximab. Adalimumab, ibritumomab tiuxetan, infliximab, and naxitamab-gqgk have been implicated in cases of erythema multiforme (EM). Paraneoplastic pemphigus, lichenoid dermatitis, and vesiculobullous dermatitis have occurred after rituximab. Dermatitis may occur after some mAbs, e.g., bevacizumab, catumaxomab, denosumab, and panitumumab. Immune-mediated cutaneous reactions induced by, e.g., cemiplimab-rwlc and durvalumab may be type IV hypersensitivities but mechanisms are not yet unequivocally established. Skin manifestations of rash and pruritus, often seen after many mAbs (Table 4 and Table 5), are generally not true hypersensitivity reactions.

There are a number of reports of mAb-induced cytopenias suggesting an underlying immune mechanism [19], but because of the lack of proper investigations, there are few convincing reports of the involvement of mAbs in type II hypersensitivity responses (Box 1). Thrombocytopenia after abciximab treatment [24,25] and cases of alemtuzumab-induced immune thrombocytopenia [26,27], neutropenia [27], autoimmune hemolytic anemia [28,29], and pure red cell aplasia [27] provide perhaps the best examples of immune-mediated true hypersensitivity responses. Apart from abciximab and alemtuzumab, rituximab has been implicated in thrombocytopenia [30], anemia [30], severe autoimmune hemolytic anemia [31], and early-onset and late-onset forms of neutropenia [30,32,33]. Although early- and late-onset neutropenia are well-known side effects of rituximab, the mechanisms have yet to be firmly established. Both forms are suspected examples of a mAb-induced type II hypersensitivity, although late-onset neutropenia may involve autoantibodies and appears to be due to a different mechanism than the early-onset form. Involvement of trastuzumab in severe thrombocytopenia has been reported [34]. See also the section on cytopenias below and Table 6.

**Figure 2:**
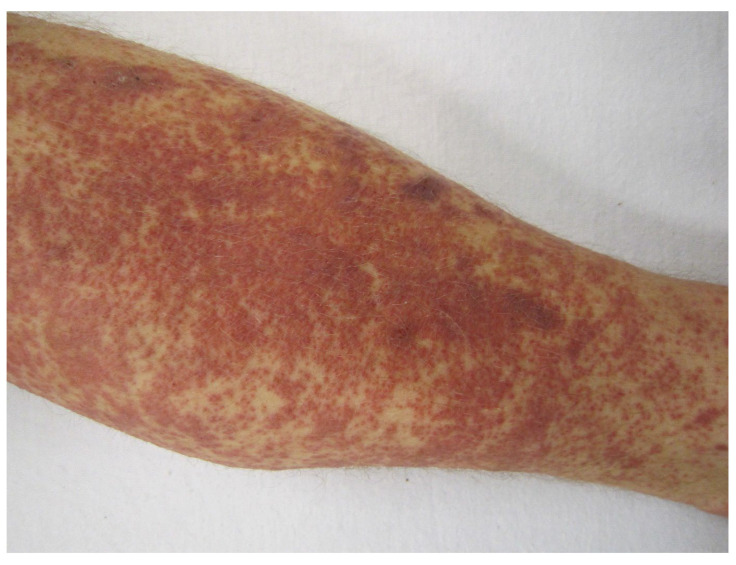
Cutaneous (hypersensitivity) vasculitis, also known as cutaneous small-vessel vasculitis and cutaneous leukocytoclastic vasculitis. Author James Heilman MD. CC BY-SA 3.0 <https://creativecommons.org/licenses/by-sa/3.0>, via Wikimedia Commons (accessed on 14 December 2021).

Hypersensitivity (cutaneous) vasculitis (Figure 2), serum sickness, and hypersensitivity pneumonitis are examples of **type III hypersensitivities** induced by mAbs (Box 1, Table 5). Apart from the fully human mAbs adalimumab and alirocumab (the latter subject to a warning), for possible hypersensitivity vasculitis, again, the chimeric antibodies, such as rituximab and infliximab, are the biggest cause of reactions. For example, cutaneous vasculitis associated with infliximab in the treatment of rheumatoid arthritis is known [35], and there are a number of reports of rituximab-induced vasculitis [36,37] and serum sickness [38,39,40]. In fact, rituximab-induced serum sickness is said to occur in up to 20% of treated patients [41]. Checkpoint inhibitors ipilimumab, nivolumab, pembrolizumab, cemiplimab-rwlc, atezolizumab, avelumab, and durvalumab (Table 5) may cause hypersensitivity pneumonitis, generally thought to be a combined type III and IV hypersensitivity in a Th1/Th17 response [42,43,44]. As well as the adverse pulmonary reactions (Table 5 and Table 6), the checkpoint inhibitors may also provoke immune-mediated colitis, endocrinopathies, hepatitis, nephritis, and thyroiditis, reactions that might involve a type III hypersensitivity mechanism (Table 6).

**Figure 3:**
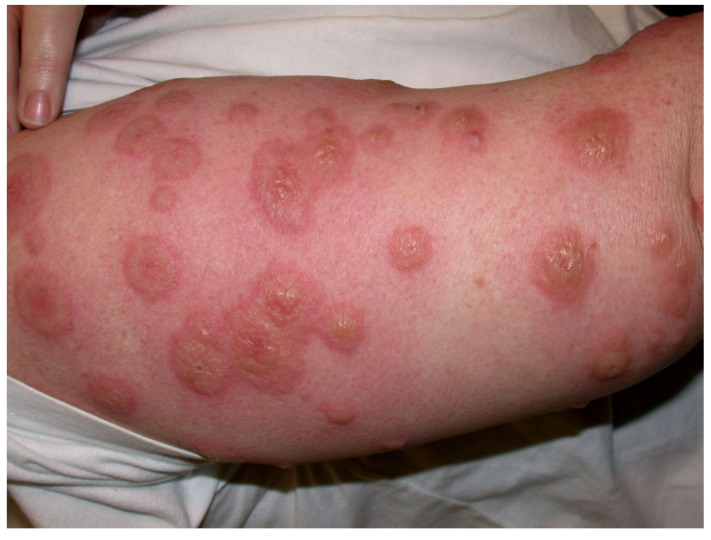
Erythema multiforme with circumscribed bullous lesions. Image courtesy of Dr Adrian Mar.

Almost 40% of the 110 approved mAbs are associated with some sorts of adverse cutaneous effects, including **type IV hypersensitivities** [19] with rare cases of life-threatening cutaneous toxidermias (Table 4, Table 5 and Table 6, Box 1). Ibritumomab has an FDA boxed warning for severe cutaneous and mucocutaneous reactions, which includes Stevens–Johnson syndrome (SJS), toxic epidermal necrolysis (TEN), erythema multiforme (EM) (Figure 3), exfoliative dermatitis, and bullous dermatitis. Warnings and precautions apply to brentuximab vedotin for SJS; rituximab has been involved in cases of SJS, TEN, paraneoplastic pemphigus, lichenoid dermatitis, and vesiculobullous dermatitis; and EM has occurred with naxitamab-gqgk therapy. EM, SJS, and psoriasis have been reported for adalimumab. EGFR-targeted mAbs are known for so-called dermatologic acneiform toxicities that are not immune-mediated (see below, Section 4.2, Cutaneous reactions).

### 4.2. Non-Immune-Mediated Adverse Responses to Approved Monoclonal Antibodies

As mentioned above, despite the specific targeting of mAbs to a particular disease-/disorder-associated tissue(s), the range and number of adverse events during or following therapy can sometimes be large and diverse. As summarized and discussed in Table 4, Table 5 and Table 6 and Box 2, many of these events do not have an immune basis or such a basis has yet to be convincingly demonstrated, either because sufficient investigation has yet to be undertaken or because of the clinical and laboratory difficulties involved in defining a precise mechanism(s). The list of recorded mAb-induced non-immune events is extensive and includes injection site reactions, infusion reactions, cytopenias, lung and liver injuries, heart effects, dermatologic toxicities, embryo and fetal toxicities, and a number of potentially life-threatening syndromes occurring with low frequency (Table 6, Box 2). It should be pointed out, however, that within some of these categories, it might be argued that there is, or may be, an immunological component with the involvement of cells and/or cytokines normally present in many inflammatory and immunological reactions.

**Injection site reactions** are very common, and when the preferred terms used in the Federal Adverse Event (FAERS) reporting system to describe such reactions are considered, namely, irritation, erythema, rash, bruising, swelling, induration, extravasation, reactions, pruritus, urticaria, hemorrhage, hematoma, and pain, it becomes apparent as to why such patient reactions are seen so regularly. In any injected population, it is to be expected that at least some individuals will respond with at least one of the above adverse effects. In the post-marketing period, the larger the population injected, the wider the collective list of adverse effects seen. The preferred terms listed are from the Medical Dictionary for Regulatory Activities (MedDRA; http://www.meddra.org/ accessed on 14 December 2021). Of the 66 approved mAbs for non-cancer therapy surveyed here, the FDA in its warnings, precautions, and lists of adverse reactions mentions injection site reactions as an adverse event for 24 (≈37%).

Box 2 **Non-immune-mediated adverse events to monoclonal antibodies (mAbs).**

- Infusion reactions. Usually mild–moderate or controllable by premedication. Fatal reactions can occur. Reactions have been recorded for almost 50% of approved mAbs. FDA boxed warnings for infusion reactions apply to 8 mAbs used for cancer therapy and 1 mAb used for other therapies.
- Cytopenia: Mechanisms of mAb-induced thrombocytopenia, neutropenia, lymphopenia, and hemolytic anemia are often not investigated/established. Cytopenia seen in more than 30% of the mAbs, especially those used in cancer therapy. Some may be immune-mediated.
- mAb-induced lung disease: Pathogenesis and pathophysiology are generally not known. At least 21 mAbs implicated. Some reactions are known, or suspected, to be immune-mediated.
- Cardiac events: Mechanisms mostly obscure. At least 20 mAbs implicated.
- Liver events: At least 22 mAbs implicated. Immune-mediated hepatitis is seen but other mechanisms often not well understood.
- Dermatologic toxicities: 39 (≈36%) of the mAbs elicited adverse cutaneous reactions of different severity from mild to severe. Rash and/or pruritus are common and were not included in the assessments. Apart from severe toxidermias (see text), papulopustular (acneiform) skin eruptions occur in response to EGFR-targeted antibodies, in particular, cetuximab, necitumumab, and panitumumab. Adverse reactions were seen in ≈29% of the non-cancer group and 50% of the mAbs used for cancer therapy.
- Embryo-fetal toxicity is recognized for 27 (≈25%) of the mAbs, including eight antibody–drug conjugates.
- Cytokine release syndrome (CRS): The distinguishing features between CRS and infusion reactions are often not clear. mAbs implicated include blinatumomab and catumaxomab.
- Tumor lysis syndrome (TLS): Anti-cancer mAbs may destroy large numbers of cells in a short period of time. Seen with brentuximab vedotin, blinatumomab, rituximab, and polatuzumab vedotin-piiq.
- Progressive multifocal leukoencephalopathy (PML): Rare but occasionally seen after mAbs directed to B cells, e.g., brentuximab vedotin, rituximab, obinutuzumab, vedolizumab, polatuzumab vedotin-piiq, and natalizumab.
- Other syndromes of poorly understood pathogenesis: Reversible posterior leukoencephalopathy syndrome (RPLS) ^1^ (cases reported after, e.g., bevacizumb and ramucirumab); immune reconstitution inflammatory syndrome (IRIS) (natalizumab); systemic inflammatory response syndrome (SIRS) (catumaxomab, eculizumab); capillary leak syndrome (CLS) (bevacizumab, dinutuximab); macrophage activation syndrome (MAS) (canakinumab).

^1^ Also known as posterior reversible encephalopathy syndrome (PRES)

**Infusion reactions** [3,19] to mAbs are common, usually with mild to moderate ‘flu’-like symptoms, but serious, potentially fatal reactions can occur. Table 6 shows that infusion reactions are known for 53 of the 110 approved mAbs (Table 4 and Table 5). Reactions may resemble anaphylaxis, and hypotension, cardiac arrest, urticaria, rash and pruritus may occur, usually after the first or second infusion, but IgE antibody reactions generally have a faster onset (often within minutes) and effects are more severe. The cytokines tumor necrosis factor (TNF) and IL-6, as well as high counts of circulating lymphocytes (e.g., >50 × 10^9^/L) are thought to be involved [45]. The highest incidence of reactions occurs with human–rodent chimeric antibodies, e.g., rituximab and infliximab, and some humanized mAbs such as alemtuzumab, ocrelizumab, and trastuzumab. Table 6 lists the 53 mAbs shown to provoke infusion reactions. Rituximab and trastuzumab show the highest incidence of reactions with incidences for first infusion reactions of ≈77% and ≈40%, respectively. Premedication may be necessary in order to avoid or lessen reactions, for example, as sometimes found necessary with elotuzumab infused for multiple myeloma [46]. Overall, mAbs involved show a two to one infusion reaction ratio of mAbs for cancer compared to those for other indications. Eight mAbs for cancer indications carry a black box warning for infusion reactions, while 22 are subject to a warnings and precautions notice. The corresponding warnings for mAbs used for non-cancer therapies are one and nine, respectively.

**Cytopenias** commonly occur during and/or following mAb therapy, especially as a result of anti-cancer therapies. Of 34 mAbs implicated in the induction of cytopenias, 24 (≈71%) are anti-cancer agents and 10 (≈29%) relate to other indications (Table 4, Table 5 and Table 6). FDA boxed warnings have been issued for three mAbs, namely, for sacituzumab govetican-hziy-induced severe neutropenia and for cytopenia following ibritumomab tiuxetan and alemtuzumab, while FDA general warnings and precautions apply to 21 other mAbs listed in Table 7. In addition, other warnings of adverse events apply to brodalumab for neutropenia; to tocilizumab for neutropenia and thrombocytopenia; and to different cytopenias, namely, lymphocytopenia, for a high proportion of anti-neoplastic mAbs (Table 4 and Table 5). Note that because mechanisms of mAb-induced thrombocytopenia, neutropenia, lymphocytopenia, anemia, and what is often simply termed ‘cytopenia’ are often not investigated, some events may, in fact, be immune-mediated.

Mab-induced **pulmonary adverse events** comprise a heterogeneous group of disorders, many of which remain poorly understood mechanistically. Of the 21 mAbs (counting alemtuzumab as Lemtrada^®^ and Campath^®^ as one mAb) listed in Table 6 and Box 3 (see also Table 4 and Table 5), immune-mediated or hypersensitivity pneumonitis is recognized as an important adverse event for an increasing number of mAbs, particularly checkpoint inhibitors [42,43,44,47]. This condition is now considered to be a combined type III and IV hypersensitivity in a Th1/Th17 response. Pneumonitis associated with checkpoint inhibitors is a rare, potentially fatal immune disease with an incidence of 2–5% [48]. Interestingly, the incidence is higher in non-small cell lung cancer than in melanoma [49]. For rituximab, while early-onset organizing pneumonia may be a hypersensitivity reaction, its prognosis is poorer than the late-onset form [50], which may be either a toxicity or due to immune restoration. Acute respiratory distress syndrome (ARDS) [51], seen for example with rituximab, trastuzumab, and ado-trastuzumab, may result from the release of pro-inflammatory cytokines such as IL-1β, TNF-α, IL-6, and IL-8, which are elevated both in bronchoalveolar lavage fluid and circulating plasma in ARDS patients [52]. Rituximab, alemtuzumab, trastuzumab, and panitumumab are responsible for the most severe and widest range of adverse lung events (Box 3).

Adverse **cardiac events** have occurred with at least 20 of the 110 approved mAbs (Table 4, Table 5 and Table 6 and Table 8) in a range of effects, including cardiomyopathy, myocardial infarction, cardiac arrhythmias, cardiopulmonary arrest, congestive heart failure, left ventricular dysfunction (LVD), decreased left ventricular ejection fraction (LVED), and QT interval prolongation (Table 8). FDA black box warnings apply to necitumumab for cardiopulmonary arrest; romosozumab-aqqg for the risk of myocardial infarction, cardiac events, stroke, and cardiovascular death; aldo-trastuzumab emtansine for cardiac toxicity; margetuximab-cmkb for LVD; and pertuzumab and trastuzumab, each for cardiomyopathy. Patients given mAbs targeted to HER2, namely, trastuzumab, ado-trastuzumab emtansine, and pertuzumab, show an increased risk of decreased LVED, especially if also given anthracyclines. Trastuzumab increases the risk of myocardial infarction 4–6 times, and again, the risk is highest when anthracyclines are also administered. Fam-trastuzumab deruxtecan-nxki carries a warning for LVD. Kounis and coworkers [53] believe that it is likely that many of the cardiac toxicities associated with mAbs used in cancer therapy share the same pathophysiology with Kounis syndrome. Suggested possible mAb involvements include -ximabs (e.g., rituximab, cetuximab, brentuximab); -zumabs (alemtuzumab, bevacizumab, trastuzumab, pertuzumab); -umabs (ipilimumab, panitumumab); and -omabs (catumaxomab, ibritumomab).

Box 3 **Pulmonary adverse events caused by approved monoclonal antibodies.**

- Mouse antibodies
  ߋ Ibrutumomab tiuxetan: hypersensitivity bronchospasm
- Human-mouse chimeric antibodies
  ߋ Cetuximab: interstitial pneumonitis
  ߋ Infliximab: interstitial lung disease
  ߋ Rituximab: ARDS, BOOP, bronchospasm, diffuse alveolar hemorrhage, immune-mediated (hypersensitivity) pneumonitis
- Humanised antibodies
  ߋ Ado-trastuzumab: interstitial lung disease, pneumonitis, ARDS, dyspnea, pulmonary infiltrates, radiation pneumonitis
  ߋ Alemtuzumab: pneumonitis, bronchospasm, diffuse alveolar hemorrhage, pulmonary infection
  ߋ Amivantamab-vmjw: interstitial lung disease, pneumonitis
  ߋ Atezolizumab: immune-mediated pneumonitis, dyspnea
  ߋ Bevacizumab: anaphylaxis/bronchospasm, pulmonary hemorrhage from tumor site
  ߋ Dostarlimab-gxly: immune-mediated pneumonitis
  ߋ Fam-trastuzumab deruxtecan-nxki: interstitial lung disease, pneumonitis
  ߋ Pembrolizumab: immune-mediated pneumonitis, dyspnea
  ߋ Trastuzumab: ARDS, BOOP, dyspnea, interstitial pneumonitis, pleural effusions, pulmonary infiltrates/fibrosis/edema

- Fully human antibodies
  ߋ Adalimumab: interstitial lung disease
  ߋ Avelumab: immune-mediated pneumonitis
  ߋ Cemiplimab-rwlc: immune-mediated pneumonitis
  ߋ Durvalumab: immune-mediated pneumonitis, dyspnea
  ߋ Golimumab: interstitial lung disease
  ߋ Ipilimumab: immune-mediated pneumonitis
  ߋ Nivolumab: immune-mediated pneumonitis, dyspnea
  ߋ Panitumumab: interstitial lung disease, lung infiltrates, pneumonitis, pulmonary fibrosis
  ߋ Tisotumab vedotin-tftv: pneumonitis

ARDS—acute respiratory distress syndrome; BOOP—bronchiolitis obliterans organizing pneumonia; ‘pneumonitis’ is used when the mechanism remains uncertain.

As occurs with mAb-induced pulmonary adverse events, checkpoint inhibitors, both PD-L1- and PD-l-targeted mAbs, may elicit immune **adverse reactions in the liver** in the form of immune-mediated hepatitis. Another immune-based adverse effect may occur with the CD25 (IL-2R α-chain)-targeted mAb daclizumab, which is subject to an FDA box warning for hepatic injury including via an autoimmune mechanism. Other mAb-provoked adverse liver injuries include direct toxicities and reactivation of hepatitis (Table 4, Table 5 and Table 6 and Table 9). FDA warnings and precautions for non-immune mAb-induced liver injury apply to adalimumab, certolizumab pegol, evolocumab, golimumab, infliximab, natalizumab, vedolizumab, brentuximab vedotin, catumaxomab, cemiplimab-rwlc, elotuzumab, ofatumumab, polatuzumab vedotin-piiq, and rituximab. Four mAbs are subject to boxed warnings, gemtuzumab ozogamicin and inotuzumab ozogamicin for hepatotoxicity, including severe or fatal hepatic veno-occlusive disease; ado-trastuzumab emtansine for hepatotoxicity; and obinutuzumab for hepatitis B reactivation (Table 9). Three of these four mAbs are antibody–drug conjugates, suggesting involvement of the attached toxin in the severe hepatotoxicities. A warning applies to satralizumab-mwge for elevated liver enzymes ALT and AST.

**Figure 4:**
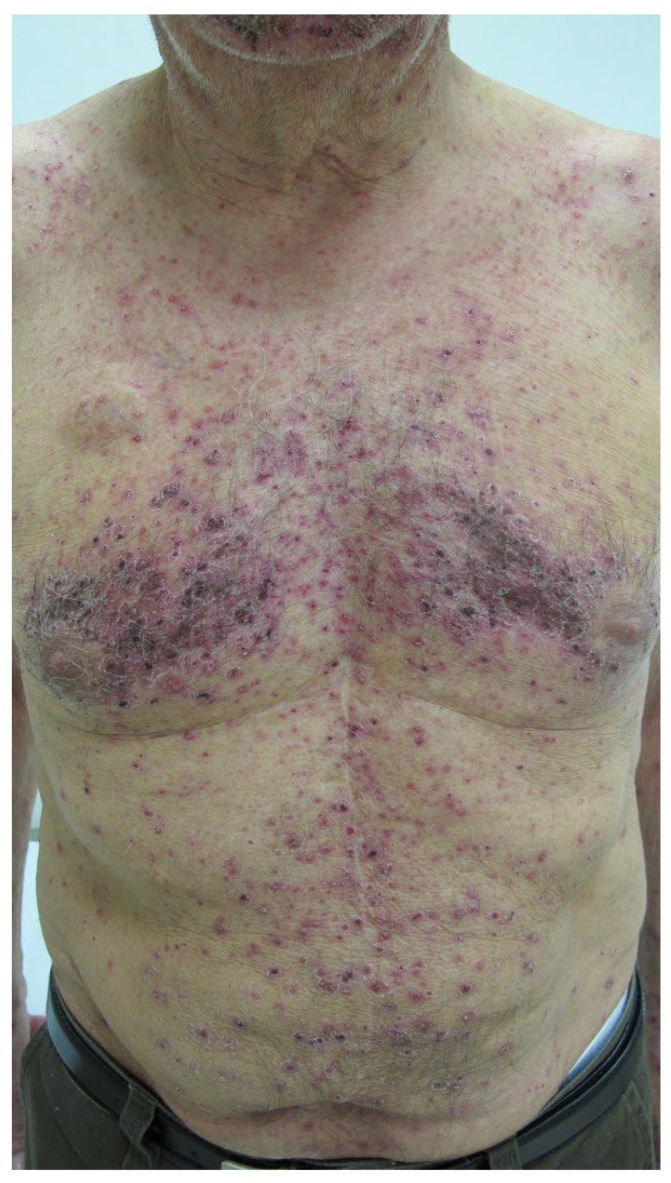
Papulopustular (acneiform) eruption on a patient during treatment with panitumumab, targeted to epidermal growth factor receptor (EGFR). From Fabbrocini, G.; Cameli, N.; Romano, M.C.; et al. Chemotherapy and skin reactions. *J. Exp. Clin. Cancer Res.* **2012**, *31*, 50. DOI: 10.1186/1756-9966-31-50 [54], an Open Access article distributed under the terms of the Creative Commons Attribution License (http://creativecommons.org/licenses/by/2.0) (accessed on 14 December 2021).

**Cutaneous reactions** have been associated with at least 39 of the 110 different mAbs (≈36%; counting alemtuzumab and denosumab each only once) (Table 6). As discussed above, some of these reactions are true type IV hypersensitivities, and there are a few recorded examples of type I reactions such as urticaria (e.g., alirocumab), but mechanisms remain to be established for many of the other adverse events (Table 4 and Table 5, Box 2). FDA warnings and precautions for dermatologic toxicity/reactions have been issued for cemiplimab-rwlc, cetuximab, denosumab, dostarlimab-gxly, durvalumab, enfortumab vedotin-ejfv, and mogamulizumab-kpkc. It is not clear whether or not at least some of the skin reactions following checkpoint inhibitors (cemiplimab-rwlc, dostarlimab-gxly, durvalumab) are immune-mediated. Skin reactions to loncastumab tesirine-lpyl may also demonstrate photosensitivity. Antibodies targeted to EGFR, namely, amivantamab-vmjw, cetuximab, necitumumab, and panitumumab, are known to produce, and are subject to, FDA warnings for papulopustular (acneiform) skin eruptions (Figure 4) [54] and mucocutaneous reactions (mucositis, xerosis, paronychia, fissures, palmar-plantar rash, skin hyperpigmentation, and others), both of which are not immune-mediated. In addition, panitumumab carries an FDA black box warning for dermatologic toxicity and has been implicated in cases of erythema, exfoliation, paronychia, skin fissures, photosensitivity, xerosis, and pruritus.

### 4.3. Rare Syndromes Associated with Monoclonal Antibody Therapy

Some rare, potentially life-threatening syndromes (Box 4) may occur with low frequency following the administration of some mAbs. **Cytokine release syndrome (CRS)** [55] shows similarities to severe infusion reactions in that both are related to a high lymphocyte count; counts greater than 50 × 10^9^/L are associated with CRS and the release of TNF and IL-6. Symptoms include fever, chills, hypotension, nausea, vomiting, dyspnea, and an increase in liver enzymes. Rituximab is a well-known cause of CRS; other implicated mAbs are alemtuzumab, blinatumomab, and catumaxomab. **Hemophagocytic lymphohistiocytosis (HLH)** [56] is a rare, highly inflammatory disorder resembling cytokine storm involving proliferation of activated T cells and macrophages with the release of large amounts of cytokines, particularly IFN gamma, TNF, and GM-CSF. IL-1 and IL-6 released from activated macrophages are responsible for the inflammatory response, tissue damage, and symptoms of HLH. Two forms of HLH are known, primary, or familial, HLH and secondary, or acquired, HLH that occurs after malignancy, infection, or immunodeficiency. Blinatumomab is well known to be a rare cause and, more recently, immune checkpoint inhibitors avelumab, ipilimumab, and nivolumab have been rarely implicated. In the **immune reconstitution inflammatory syndrome (IRIS)** [57], also called immune recovery syndrome, restoration of immunity is, paradoxically, accompanied by deterioration of a known or new condition. Examples of the syndrome are seen in AIDS and tuberculosis. The pathogenesis of the condition is poorly understood. MAbs implicated in IRIS are adalimumab, ibalizumab-uiyk, infliximab, and natalizumab. **Macrophage activation syndrome (MAS)** [58] resembles HLH, but the name is traditionally reserved for the HLH-like inflammatory reaction seen in at least 10% of patients with rheumatologic diseases, in particular systemic juvenile idiopathic arthritis (SJIA). MAS, which can be rapidly fatal, is mediated by an uncontrolled proliferation of T cells and macrophages exhibiting hemophagocytic activity [59]. MAbs known to precipitate the syndrome include alemtuzumab, canakinumab, and tocilizumab. **Progressive multifocal leukoencephalopathy (PML)** [60] is a rare, usually fatal demyelinating disease characterized by inflammation and progressive brain damage. It is caused by infection with the normally harmless JC virus that becomes lethally active in immunosuppressed patients, in some autoimmune diseases, and in patients receiving chemotherapy, including some biologics. MAbs involved include belimumab, brentuximab vedotin, infliximab, eculizumab, natalizumab, ofatumumab, polatuzumab vedotin-piiq, rituximab, and vedolizumab. In **reversible posterior encephalopathy syndrome (RPLS)**, also called posterior reversible encephalopathy syndrome (PRES [61]), edematous changes occur in the brain perhaps as a result of systemic hypertension leading to hypoxia and vasogenic edema. However, some cases of RPLS appear to occur in the absence of hypertension and others in the absence of inflammation. MAbs associated with RPLS include bevacizumab, certolizumab pegol, infliximab, dinutuximab, naxitamab-gqgk, ramucirumab, rituximab, and ustekinumab. **Systemic capillary leak syndrome (SCLS)** [62], also known simply as capillary leak syndrome, vascular leak syndrome, and Clarkson’s disease, has symptoms of body weight increase, malaise, weakness, pyrexia, myalgia, abdominal pain/vomiting, and diarrhea. An increase in vascular permeability and extravasation of fluids leads to peripheral and interstitial edema and, in severe form, pulmonary and cardiovascular failure. MAbs reported to be associated with CLS include alemtuzumab, basiliximab, bevacizumab, catumaxomab, dinutuximab, the immune checkpoint inhibitor nivolumab, and rituximab. **Systemic inflammatory response syndrome (SIRS)** [63], related to sepsis, can cause organ dysfunction and failure. It may be caused by infection or have a noninfectious basis such as trauma, pancreatitis, ischemia, anaphylaxis, or treatment with a biologic agent. The condition proceeds via activation of an inflammatory cascade of cytokines including TNF; IFN gamma; and IL-1, -6, and -8. SIRS has been reported following catumaxomab and eculizumab. **Tumor lysis syndrome (TLS)** [64] occurs most often in patients with leukemia and high-grade lymphomas where there are large numbers of cancer cells. Death of the cells results in marked ionic imbalance due to hypercalcemia, hyperkalemia, hyperphosphatemia, and hyperuricemia. This can lead to renal failure, cardiac arrhythmias, seizures, and death. The mAbs most often associated with TLS are alemtuzumab, blinatumomab, brentuximab vedotin, ipilimumab, obinutuzumab, polatuzumab vedotin-piiq, and rituximab (Box 4).

Box 4 **Monoclonal antibodies associated with rare syndromes.**

- **Cytokine release syndrome (CRS)**
  Alemtuzumab; blinatumomab; catumaxomab; rituximab
- **Hemophagocytic lymphohistiocytosis (HLH)**
  Alemtuzumab; avelumab; blinatumomab; ipilimumab; nivolumab
- **Immune reconstitution inflammatory syndrome (IRIS)**
  Adalimumab; ibalizumab-uiyk; infliximab; natalizumab
- **Macrophage activation syndrome (MAS)**
  Alemtuzumab; canakinumab; tocilizumab
- **Progressive multifocal leukoencephalopathy (PML)**
  Belimumab; brentuximab vedotin; infliximab; eculizumab; natalizumab; ocrelizumab; ofatumumab; polatuzumab vedotin-piiq; rituximab; vedolizumab
- **Reversible posterior encephalopathy syndrome (RPLS)**
  Bevacizumab; certolizumab pegol; infliximab; dinutuximab; naxitamab-gqgk; ramucirumab; rituximab; ustekinumab
- **Systemic capillary leak syndrome (SCLS)**
  Alemtuzumab; basiliximab; bevacizumab; catumaxomab; dinutuximab; nivolumab; rituximab
- **Systemic inflammatory response syndrome (SIRS)**
  Catumaxomab; eculizumab
- **Tumor lysis syndrome (TLS)**
  Alemtuzumab; blinatumomab; brentuximab vedotin; ipilimumab; obinutuzumab; polatuzumab vedotin-piiq; rituximab

## 5. Concluding Remarks

**Figure 5:**
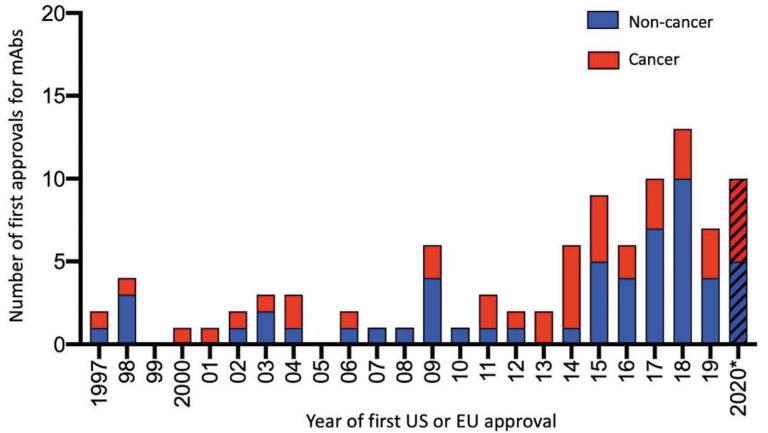
Numbers of mAbs approved by the FDA and/or EMA during the 24 year period 1997–2020. Biosimilar and Fc fusion proteins are not included. Note that in 2021, 14 mAb products were approved. *: Data publicly available as of 25 November 2020. From Kaplon, H.; Reichert, J.M. Antibodies to watch in 2021. *Mabs* **2021**, *13*, e1860476, doi.org/10.1080/19420862.2020.1860476 [65], an Open Access article distributed under the terms of the Creative Commons Attribution-NonCommercial License (http://creativecommons.org/licenses/by-nc/4.0) (accessed on 14 December 2021).

At the beginning of 2022, the catalog of mAbs approved for therapy by the FDA and/or EMA consisted of 66 approved for non-cancer indications and 46 for cancer therapy. Unsurprisingly because of their clinical success, the number of approved mAbs continues to expand, for example, in the 17 year period 1997–2013, 34 mAbs were approved, whereas in the 7 years of 2014–2020, the approved total was 61 (Figure 5) [65]. From 1997 until the present time (December 2021), 110 mAbs have received approval from the FDA and/or EMA (Figure 5). In 2021, 14 products were approved: aducanumab, amivantamab, anifrolumab, bimekizumab, casirivimab + imdevimab; dostarlimab-gxly, efgartigimod-alfa-fcab, evinacumab, loncastuximab teserine-lpyl, regdanvimab, sotrovimab, tezepelumab-ekko, tisotumab vedotin, and tralokinumab. Approved by the EMA, casirivimab + imdevimab, regdanvimab, and sotrovimab are the first three preparations for the treatment of COVID-19, each targeted to the spike protein receptor-binding domain of SARS-CoV-2 (Table 1). It is clear that from information on the numbers of mAbs already undergoing clinical assessment, as well as some already marketed for other indications with the view of repurposing for the treatment of COVID-19, further approvals of mAb preparations to treat this disease are imminent [65]. In late December 2021, efgartigimod-alfa-fcab indicated for myasthenia gravis and tezepelumab-ekko for severe asthma were approved by the FDA.

In the next few years, research and clinical progress in disease pathogenesis and the identification of new disease biomarker targets, together with ongoing orphan drug development programs, will continue an inevitable expansion of the list of approved mAbs. Aspects of this expansion of great interest include a growing list of new indications; further mechanistic insights into the interplay between antibodies, cells, cytokines, chemokines, receptor interactions and downstream signaling; the appearance of new, and some unexpected, adverse events; and progress in understanding and treating such events.

## Figures and Tables

**Table 1:** Therapeutic monoclonal antibodies for non-cancer therapy currently marketed with regulatory approval from the U.S. FDA or EMA or both (as at December 2021).

Monoclonal Antibody INN and Trade Names	Antibody Type	Target	Approved Indications
**Human–Mouse Chimeric** **(-ximab)**			
Abciximab (ReoPro^®^)	Chimeric IgG Fab	Glycoprotein IIb/IIIa	Adjunct therapy for prevention of cardiac ischemic complications
Basiliximab (Simulect^®^)	Chimeric IgG1	α-chain IL-2 receptor (CD25)	Prevent organ transplant rejection
Infliximab (Remicade^®^)	Chimeric IgG1	TNF	Crohn’s disease; ulcerative colitis; RA; ankylosing spondylitis; psoriatic arthritis; plaque psoriasis
Obiltoxaximab (Anthim^®^)	Chimeric IgG1	*Bacillus anthracis* PA	Inhalational anthrax *Bacillus* *anthracis* PA
**Humanized (-zumab)**			
Alemtuzumab (Lemtrada^®^)	Humanized IgG1	CD52	Lemtrada^®^: multiple sclerosis
Benralizumab (Fasenra^®^)	Humanized IgG1 (afucosylated)	IL-5Rα	Asthma
Bimekizumab (Bimzelx^®^)	Humanized IgG1	IL-17A, IL-17F, IL-17AF	Plaque psoriasis
Brolucizumab (Beovu^®^)	Humanized single-chain (scFv) fragment	VEGF-A	Neovascular (wet) age-related macular degeneration
Caplacizumab-yhdp (Caplivi^®^)	Humanized bivalent single-domain nanobody	von Willebrand factor (vWF)	Acquired thrombotic thrombocytopenic purpura
Certolizumab pegol (Cimzia^®^)	Humanized IgG1 Fab, pegylated	TNF	Crohn’s disease; RA
Crizanlizumab-tmca (Adakveo^®^)	Humanized IgG2	P-selectin	Sickle cell disease
Daclizumab (Zinbryta^®^)	Humanized IgG2	α-chain IL-2 receptor (CD25)	Multiple sclerosis
Eculizumab (Soliris^®^)	Humanized IgG2/4	Complement C5	Paroxysmal nocturnal hemoglobinuria; atypical hemolytic uremic syndrome; neuromyelitis optica spectrum disorder (NMOSD) in adult patients who are anti-aquaporin-4 (AQP4) antibody-positive
Emicizumab-kxwh (Hemlibra^®^)	Humanized IgG4 bispecific	Factors IXa and X	Hemophilia A
Eptinezumab-jjmr (Vyepti^®^)	Humanized IgG1	CGRP	Migraine
Fremanezumab-vfrm (Ajovy^®^)	Humanized IgG4	CGRP	Migraine
Galcanezumab-gnlm (Emgality^®^)	Humanized IgG4	CGRP	Migraine
Ibalizumab-uiyk (Trogarzo^®^)	Humanized IgG4	CD4	HIV-1 infection
Idarucizumab (Praxbind^®^)	Humanized IgG1 antibody fragment Fab	Dabigatran	Reversal of anticoagulant effects of dabigatran; life-threatening or uncontrolled bleeding
Inebilizumab-cdon (Uplizna^®^)	Humanized afucosylated IgG1	CD19	Neuromyelitis optica spectrum disorder (NMOSD) in adult patients who are anti-aquaporin-4 (AQP4) antibody positive
Ixekizumab (Taltz^®^)	Humanized IgG4	IL-17A	Plaque psoriasis; psoriatic arthritis
Mepolizumab (Nucala^®^)	Humanized IgG1	IL-5	Asthma; eosinophilic granulomatosis with polyangiitis
Natalizumab (Tysabri^®^)	Humanized IgG4	α4 integrin (binds to α4β1 and α4β7 integrins)	Multiple sclerosis; Crohn’s disease
Ocrelizumab (Ocrevus^®^)	Humanized IgG1	CD20	Multiple sclerosis
Omalizumab (Xolair^®^)	Humanized IgG1	IgE	Persistent asthma; chronic idiopathic urticaria
Palivizumab (Synagis^®^)	Humanized IgG1	RSVF	Prevention of lower respiratory tract disease RSV in children
Ranibizumab (Lucentis^®^)	Humanized IgG1 Fab	VEGF-A	Neovascular (wet) age-related macular degeneration; macular edema following retinal vein occlusion; diabetic macular edema
Ravulizumab-cwvz (Ultomiris^®^)	Humanized IgG2/4	Complement C5	Paroxysmal nocturnal hemoglobinuria
Reslizumab (Cinqair^®^)	Humanized IgG4	IL-5	Asthma
Risankizumab-rzaa (Skyrizi^®^)	Humanized IgG1	IL-23 p19	Plaque psoriasis
Romosozumab-aqqg (Evenity^®^)	Humanized IgG2	Sclerostin	Osteoporosis
Satralizumab-mwge (Enspryng^®^)	Humanized IgG2	IL-6R	Neuromyelitis optica spectrum disorder (NMOSD) in adult patients who are anti-aquaporin-4 (AQP4) antibody-positive
Tildrakizumab-asmn (Ilumetri^®^; Ilumya^®^)	Humanized IgG1	IL-23 p19	Plaque psoriasis
Tocilizumab (Actemra^®^; RoActemra^®^)	Humanized IgG1	IL-6R	RA; polyarticular juvenile idiopathic arthritis; systemic juvenile idiopathic arthritis
Vedolizumab (Entyvio^®^)	Humanized IgG1	α4β7 integrin	Adult ulcerative colitis; adult Crohn’s disease
**Fully human (-umab)**			
Adalimumab (Humira^®^)	Human IgG1	TNF	RA; psoriatic arthritis; ankylosing spondylitis; plaque psoriasis; Crohn’s disease
Aducanumab-avwa (Aduhelm^®^)	Human IgG1	Amyloid beta	Alzheimer’s disease
Alirocumab (Praluent^®^)	Human IgG1	PCSK9	Heterozygous FH; atherosclerotic CV disease requiring additional ↓ of LDL-C
Anifrolumab-fnia (Saphnelo^®^)	Human IgG1	Subunit I type I interferon receptor (IFNAR)	Systemic lupus erythematosis
Ansuvimab-zykl (Ebanga^®^)	Human IgG1	*Zaire ebolavirus* (EBOV) glycoprotein 1 (GP1)	*Zaire ebolavirus* infection
Atoltivimab, Maftivimab and Odesivimab-ebgn (Inmazeb^®^)	Human IgG1	*Zaire ebolavirus* (EBOV) glycoprotein 1 (GP1)	*Zaire ebolavirus* infection
Belimumab (Benlysta^®^)	Human IgG1	BlyS	Systemic lupus erythematosus
Bezlotoxumab (Zinplava^®^)	Human IgG1	*Clostridium difficile* toxin B	Recurrence of *Clostridium difficile* toxin B infection
Brodalumab (Siliq^®^; Kyntheum^®^; Lumicef^®^)	Human IgG2	IL-17RA	Plaque psoriasis
Burosumab-twza (Crysvita^®^)	Human IgG1	FGF23	X-linked hypophosphatemia
Canakinumab (Ilaris^®^)	Human IgG1	IL-1β	Cryopyrin-associated periodic syndromes (CAPS) including familial cold autoinflammatory and Muckle–Wells syndromes; SJIA with body weight ≥7.5 kg; NOMID/CINCA; FCAS/FCU; gouty arthritis
Casirivimab + Imdevimab (REGEN-COV^®;^ Ronapreve^®^)	Human IgG1	The 2 mAbs bind to separate epitopes of the spike protein RBD of SARS-CoV-2, thus preventing its binding to the human ACE2 receptor and subsequent cell entry	COVID-19 disease
Denosumab (Prolia^®^)	Human IgG2	RANKL	Bone loss—for osteoporosis and to increase bone mass in menopausal women at high risk of fracture
Dupilumab (Dupixent^®^)	Human IgG4	IL-4Rα subunit	Atopic dermatitis
Efgartigimod-alfa-fcab (Vyvgart^®^)	Human IgG1 Fc fragment	Neonatal Fc receptor FcRn	Generalized myasthenia gravis
Emapalumab-lzsg (Gamifant^®^)	Human IgG1	IFNγ	HLH
Erenumab-zooe (Aimovig^®^)	Human IgG2	CGRP receptor	Migraine
Evinacumab-dgnb (Evkeeza^®^)	Human IgG4	ANGPTL3 (angiopoietin-like 3)	Homozygous familial hypercholesterolemia (HoFH)
Evolocumab (Repatha^®^)	Human IgG2	PCSK9	Primary hyperlipidemia and mixed dyslipidemia; homozygous FH to reduce LDL-C and other lipids
Golimumab (Simponi^®^)	Human IgG1	TNF	RA; psoriatic arthritis (both in combination with methotrexate); ankylosing spondylitis
Guselkumab (Tremfya^®^)	Human IgG1	IL-23	Plaque psoriasis
Lanadelumab-flyo (Takhzyro^®^)	Human IgG1	Plasma kallikrein	HAE prevention
Raxibacumab (ABthrax^®^)	Human IgG1	*Bacillus anthracis* PA	Inhalational anthrax to *Bacillus anthracis* and prophylaxis in absence of alternative therapies
Regdanvirimab (Regkirona^®^)	Human IgG1	mAb binds to the spike protein RBD of SARS-CoV-2 preventing its binding to the human ACE2 receptor and subsequent cell entry	COVID-19 disease
Sarilumab (Kevzara^®^)	Human IgG1	IL-6R	RA
Secukinumab (Cosentyx^®^)	Human IgG1	IL-17A	Moderate to severe plaque psoriasis
Sotrovimab (Xevudy^®^) ^1^	Human IgG1	Spike protein RBD of SARS-CoV-2	COVID-19 disease
Teprotumumab-trbw (Tepezza^®^)	Human IgG1	IGF-1R	Thyroid eye disease
Tezepelumab-ekko (Tezspire^®^)	Human IgG2	Thymic stromal lymphopoietin	Severe asthma
Tralokinumab (Adtralza^®^)	Human IgG4	IL-13	Atopic dermatitis
Ustekinumab (Stelara^®^)	Human IgG1	IL-12, IL-23	Plaque psoriasis

ACE2—angiotensin-converting enzyme 2; ADCC—antibody-dependent cell-mediated cytotoxicity; BlyS—B lymphocyte stimulator, also known as B cell-activating factor, BAFF; C5—complement component 5; CDC—complement-dependent cytotoxicity; CGRP—calcitonin gene-related peptide; CHO—Chinese hamster ovary cells; CINCA—chronic infantile neurological, cutaneous, articular syndrome; COVID—coronavirus disease; CV—cardiovascular; EMA—European Medicines Agency; FCAS—familial cold autoinflammatory syndrome; FCU—familial cold urticaria; FDA—U.S. Food and Drug Administration; *FH*—familial hypercholesterolemia; FGF23—fibroblast growth factor 23; GI—gastrointestinal; *HAE*—hereditary angioedema; HIV—human immunodeficiency virus; *HLH*—primary hemophagocytic lymphohistiocytosis; IGF-1R—insulin-like growth factor-1 receptor; IPP—International Nonproprietary Name; LDL—low-density lipoprotein; LDL-C—LDL-cholesterol; LDLR—LDL receptor; NLRP-3—gene cryopyrin or nucleotide-binding domain, leucine rich family, pyrin domain-containing 3 gene; NOMID—neonatal-onset multisystem inflammatory disease; NSCLC—non-small cell lung cancer; NSO—non-Ig-secreting, non-L chain-synthesizing, 8-azaguanine-resistant and HAT-sensitive mouse myeloma cell line; PA—protective antigen of *B. anthracis* toxin; PCSK9—proprotein convertase subtilisin/kexin type 9; RA—rheumatoid arthritis; RANKL—receptor activator of nuclear factor kappa-B ligand (CD254), a member of the TNF cytokine family; RBD—receptor-binding domain; RSV—human respiratory syncytial virus (*F* viral protein coat antigen); SARS-CoV-2—severe acute respiratory syndrome coronavirus 2; SJIA—active systemic juvenile idiopathic arthritis; Sp2/0—BALB/c mouse spleen cells fused with P3 myeloma. Cells do not secrete Ig, are resistant to 8-azaguanine, and are HAT-sensitive; TNF— tumor necrosis factor; VEGF—vascular endothelial growth factor (a subfamily of growth factors; includes VEGF-A); VEGFR2—vascular endothelial growth factor receptor 2, also known as KDR (kinase insert domain-containing receptor), FLK1 (fetal liver kinase 1), or CD309. ^1^ Note added in press: Approved by the FDA 17 December 2021. ↓ decrease.

**Table 2:** Therapeutic monoclonal antibodies for cancer therapy currently marketed with regulatory approval from the U.S. FDA or EMA or both (as at December 2021).

Monoclonal Antibody INN and Trade Name	Type of mAb	Target	Approved Indications
**Rat-mouse chimera (-axomab)**			
Catumaxomab (Removab^®^)	Rat IgG2b/Mouse IgG2a bispecfic	EpCAM/CD3	Malignant ascites
**Mouse (-omab)**			
Blinatumomab (Blincyto^®^)	Mouse scFvκ-H bispecific	CD19/CD3 epsilon	Philadelphia chromosome-negative relapsed or refractory B cell precursor acute lymphoblastic leukemia
Ibritumomab tiuxetan (Zevalin^®^)	Mouse IgG1	CD20	Non-HL
Moxetumomab pasudox– tdfk (Lumoxiti^®^)	ADC immunotoxin. Mouse single chain variable domain (scFv)	CD22	HCL
**Human-mouse chimeric** **(-ximab)**			
Brentuximab vedotin (Adcetris^®^)	Chimeric IgG1	CD30	HL after failure of stem cell transplant or chemotherapy; sALCL after failure of chemotherapy; post auto-HSCT consolidation treatment for HL
Cetuximab (Erbitux^®^)	Chimeric IgG1	EFGR	Colorectal and head and neck cancers
Dinutuximab (Unituxin^®^)	Chimeric IgG1	GD2	Pediatric patients with high-risk neuroblastoma
Isatuximab-irfc (Sarclisa^®^)	Chimeric IgG1 with 2 identical H and κ L chains	CD38	MM
Margetuximab-cmkb (Margenza^®^)	Chimeric IgG1	HER2	HER2-positive breast cancer
Rituximab (Rituxan^®^; MabThera^®^)	Chimeric IgG1	CD20	Non-HL; CLL; rheumatoid arthritis; Wegener’s granulomatosis; microscopic polyangiitis
Siltuximab (Sylvant^®^)	Chimeric IgG1	IL-6	Multicentric Castelman’s disease in patients negative for HIV and HHV-8
**Humanized (-zumab)**			
Ado-trastuzumab emtansine (Kadcyla^®^)	ADC. Humanized IgG1	HER2	HER2-positive breast cancer in patients who previously received trastuzumab or a taxane
Alemtuzumab (Campath^®^; MabCampath^®)^	Humanized IgG1	CD52	Campath, MabCampath: B cell CLL
Atezolizumab (Tecentriq^®^)	Humanised IgG1	PD-L1	MUC; NSCLC
Bevacizumab (Avastin^®^)	Humanized IgG1	VEGF-A	Metastatic colorectal cancer; non-squamous NSCLC; metastatic breast cancer; ovarian cancer; glioblastoma
Dostarlimab-gxly (Jemperli^®^)	Humanized IgG4	PD-1	Endometrial cancer
Elotuzumab (Empliciti^®^)	Humanised IgG1	SLAMF7	MM
Fam-trastuzumab deruxtecan-nxki (Enhertu^®^)	ADC. Humanised IgG1	HER2	HER2-positive breast, gastric, and GE adenocarcinomas
Gemtuzumab ozogamicin (Mylotarg^®^)	ADC. Humanized IgG4	CD33	AML
Inotuzumab ozogamicin (Besponsa^®^)	ADC. Humanized IgG4	CD22	ALL
Loncastumab tesirine-lpyl (Zynlonta^®^)	ADC. Humanized IgG1	CD19 with teserine cytotoxic agent	LBCL including DLBCL
Mogamulizumab-kpkc (Poteligeo^®^)	Humanized IgG1	CCR4	Mycosis fungoides; Sézary syndrome
Naxitamab-gqgk (Danyelza^®^)	Humanized IgG1	GD2	Neuroblastoma—antibody given in combination with GM-CSF
Obinutuzumab (Gazyva^®^; Gazyvaro^®^)	Humanized IgG1	CD20	In combination with chlorambucil for previously untreated CLL
Pembrolizumab (Keytruda^®^)	Humanized IgG4	PD-1	Unresectable or metastatic melanoma; refractory metastatic NSCLC tumors that express PD-L1
Pertuzumab (Perjeta^®^)	Humanized IgG1	HER2	Combination with trastuzumab and docetaxel for HER2-positive metastatic breast cancer
Polatuzumab vedotin-piiq (Polivy^®^)	ADC. Humanized IgG1	CD79b	Diffuse large B cell lymphoma
Sacituzumab govitecan-hziy (Trodelvy^®^)	ADC. Humanized IgG1	Trop-2 with topoisomerase inhibitor	mTNBC
Tafasitamab-cxix (Monjuvi^®^)	Humanized IgG1/2 with hybrid Fc-modified domain	CD19	DLBCL
Trastuzumab (Herceptin^®^)	Humanized IgG1	HER2	Breast cancer overexpressing HER2, metastatic gastric or GE junction adenocarcinoma overexpressing HER2
**Fully human (-umab)**			
Amivantamab-vmjw (Rybrevant^®^)	Bi-specific low fucose human IgG1-based antibody	EGFR and c-MET receptors	NSCLC
Avelumab (Bavencio^®^)	Human IgG1	PD-L1	MCC; UC; RCC
Belantamab mafodoton-blmf (Blenrep^®^)	ADC afucosylated IgG1	BCMA with MMAF microtubule inhibitor	MM
Cemiplimab-rwlc (Libtayo^®^)	Human IgG4	PD-1	CSCC
Daratumumab (Darzalex^®^)	Human IgG1	CD38	MM
Denosumab (Prolia^®^; Xgeva^®^)	Human IgG2	RANKL	Bone loss. Prolia: for osteoporosis and to increase bone mass; Xgeva: for bone metastases from solid tumors and giant cell tumor of bone
Durvalumab (Imfinzi^®^)	Human IgG1	PD-L1	UC
Enfortumab-vedotin-ejfv (Padcev^®^)	ADC human IgG1	Nectin-4 with MMAE microtubule inhibitor	UC
Ipilimumab (Yervoy^®^)	Human IgG1	CTLA-4	Metastatic melanoma
Necitumumab (Portrazza^®^)	Human IgG1	EGFR	Squamous NSCLC
Nivolumab (OPDIVO^®^)	Human IgG4	PD-1	Unresectable or metastatic melanoma and disease progression following ipilimumab and, if BRAF V600-positive, a BRAF inhibitor; NSCLC
Ofatumumab (Arzerra^®^)	Human IgG1	CD20	CLL refractory to fludarabine and alemtuzumab
Olaratumab (Lartruvo^®^)	Human IgG1	PDGFR-α	Soft tissue sarcoma
Panitumumab (Vectibix^®^)	Human IgG2	EGFR	Metastatic colorectal cancer
Ramucirumab (Cyramza^®^)	Human IgG1	VEGFR2	Gastric or GE junction adeno- carcinoma; metastatic NSCLC with docetaxel after platinum therapy; HCC; with FOLFIRI for metastatic colorectal cancer
Tisotumab vedotin-tftv (Tivdak^®^)	ADC human IgG1	TF with MMAE microtubule inhibitor	Cervical cancer

ADC—antibody drug conjugate; ALL—acute lymphoblastic leukemia; auto-HSCT—autologous hematopoietic stem cell transplantation; BRAF—proto-oncogene B-Raf; C5—complement component 5; CLL—chronic lymphocytic leukemia; CTLA-4—cytotoxic T lymphocyte-associated antigen 4 or CD152; CSCC—cutaneous squamous cell carcinoma; DLBCL—diffuse large B cell lymphoma; EGFR—epidermal growth factor receptor; EMA—European Medicines Agency; EpCAM—epithelial cell adhesion molecule; FDA—U.S. Food and Drug Administration; FOLFIRI—combination of folinic acid (leucovorin), fluorouracil, and irinotecan; GD2—glycolipid disialoganglioside on neuroblastoma, central nervous system, and peripheral nerve cells; GE—gastroesophageal; HCC—hepatocellular carcinoma; HCL—hairy cell leukemia; HER2—human epidermal growth factor receptor 2, also known as HER2/neu, ErbB2, CD340, p185, or EGFR2; HL—Hodgkin lymphoma; IPP—International Nonproprietary Name; LBCL—large B cell lymphoma; MCC—Merkel cell carcinoma; MM—multiple myeloma; MMAE—cytotoxic agent monomethyl auristatin E; MMAF—cytotoxic agent monomethyl auristatin F; mTNBC—metastatic triple-negative breast cancer; MUC—metastatic urothelial carcinoma; NSCLC—non-small cell lung cancer; PD-1—programmed cell death protein 1 or CD279; PD-L1—programmed cell death protein ligand 1; RANKL—receptor activator of nuclear factor kappa-B ligand (CD254), a member of the TNF cytokine family; RCC—renal cell carcinoma; sALCL—systemic anaplastic large cell lymphoma; teserine—also known as SG3249, a pyrrolobenzodiazepine dimer; TF—tissue factor, platelet tissue factor, factor III, CD142; Trop-2—trophoblast cell surface antigen-2; UC—urothelial carcinoma; VEGF—vascular endothelial growth factor (a subfamily of growth factors; includes VEGF-A); VEGFR2—vascular endothelial growth factor receptor 2, also known as KDR (kinase insert domain-containing receptor), FLK1 (fetal liver kinase 1), or CD309.

**Table 3:** Targets with more than one complementary approved therapeutic monoclonal antibody.

Target	Monoclonal Antibodies
	*Monoclonal antibodies for **non-cancer** therapy*
TNF	Adalimumab; certolizumab pegol; golimumab; infliximab
PCSK9	Alirocumab; evolocumab
EBOV GP1	Ansuvimab-zykl; atoltivimab; maftivimab; odesivimab-ebgn
IL-2 receptor α chain (CD25)	Basiliximab; daclizumab
VEGF-A	Brolocizumab-dbll; ranibizumab
ACE2 RBD of SARS-CoV-2	Casirivimab + imdevimab; regdanvirimab; Sotrovimab
Complement C5	Eculizumab; ravulizumab-cwvz
CGRP	Eptinezumab-jjmr; fremanezumab-vfrm; galcanezumab-gnlm
IL-17A	Ixekizumab; secukinumab
IL-5	Mepolizumab; reslizumab
α4 integrin	Natalizumab; vedolizumab
*Bacillus anthracis*	Obiltoxaximab; raxibacumab
IL-23 p19	Risankizumab-rzaa; tildrakizumab-asmn
IL-6R	Sarilumab; satralizumab-mwge; tocilizumab
	*Monoclonal antibodies for cancer therapy*
HER2	Ado-trastuzumab; fam-trastuzumab; margetuximab-cmkb; pertuzumab; trastuzumab
PD-L1	Atezolizumab; avelumab; durvalumab
PD-1	Cemiplimab-rwlc; dostarlimab-gxly; nivolumab; pembrolizumab
EGFR	Amivantamab; Cetuximab; necitumumab; panitumumab
CD38	Daratumumab; isatuximab-irfc
GD2	Dinutuximab; naxitamab-gqgk
CD20	Ibritumomab; obinutuzumab; ofatumumab; rituximab
CD22	Inotuzumab ozogamicin; moxetumomab pasudox-tdfk

For expansion of target abbreviations, see Table 1 and Table 2.

**Table 4:** Adverse events associated with approved ^1^ monoclonal antibodies used for non-cancer therapies (as at December 2021).

Monoclonal Antibody ^2^ INN and Trade Names	Target ^3^	Warnings, Precautions, Risks and Safety Concerns	Other Adverse Events ^4^, Serious and Common
Abciximab (ReoPro^®^)	Glycoprotein IIb/IIIa	Increased risk of bleeding; thrombocytopenia	*Systemic*: Bleeding; intracranial hemorrhage or stroke; GI; CV; anemia; NS; respiratory; urinary disorders *Cutaneous*: Pruritus; generalized exanthema
Adalimumab (Humira^®^)	TNF	*Boxed warning*: Serious infections; malignancy *Other*: Anaphylaxis, serious allergic reactions; hepatitis B reactivation; demyelinating disease; cytopenias; heart failure; lupus-like syndrome	*Systemic*: Infections; isr; ILD; sarcoidosis; liver failure *Cutaneous*: SJS; EM; psoriasis; cutaneous vasculitis; alopecia
Aducanumab-avwa (Aduhelm^®^)	Amyloid beta	Amyloid-related imaging abnormalities (ARIA); hypersensitivity	*Systemic*: Headache; ARIA-oedema, -headache, -H microhemorrhage, -H superficial siderosis, fall
Alemtuzumab (Lemtrada^®^)	CD52	*Boxed warning*: Autoimmunity; IRs; malignancies *Other*: Other immune cytopenias; glomerular nephropathies; thyroid disorders; delay therapy in cases of infections; pneumonitis	*Systemic*: Headache; pyrexia; nausea; UTI; herpes virus infection; extremity and back pain; dizziness; flushing; cough; chills; vomiting; dyspnea *Cutaneous*: Rash; urticaria; pruritus; dermatitis
Alirocumab (Praluent^®^)	PCSK9	Allergic reactions (pruritus, urticaria, rash) including some serious (including hypersensitivity vasculitis)	*Systemic*: Nasopharyngitis; isr; influenza; URTI; cough; sinusitis; bronchitis; diarrhea; myalgia; muscle spasms; musculoskeletal pain; liver enzyme abnormalities
Anifrolumab-fnia (Saphnelo^®^)	IFNAR	Serious infections; hypersensitivity; malignancy; avoid live attenuated vaccines and other biological therapies	*Systemic*: Nasopharyngitis; URTI; IR; bronchitis; herpes zoster; cough
Ansuvimab-zykl (Ebanga^®^)	EBOV GP1	Hypersensitivity; IR	*Systemic*: Pyrexia; tachycardia; diarrhea; vomiting; hypotension; tachypnoea; chills
Atoltivimab, Maftivimab, and Odesivimab-ebgn (Inmazeb^®^)	EBOV GP1	Hypersensitivity; IR	*Systemic*: Pyrexia; chills; tachycardia; tachypnoea; vomiting
Basiliximab (Simulect^®^)	IL-2 receptor α-chain (CD25)	*Boxed warning*: General risk of immunosuppressive therapy *Other*: Immunogenicity; hypersensitivity	*Systemic*: GI; viral infection; peripheral oedema; UTI; URTI; dyspnea; wound complications; hypertension; anemia; hypo- and hyperkalemia and hyperuricemia; headache; tremor *Cutaneous*: Rash; pruritus; hypertrichosis
Belimumab (Benlysta^®^)	BLyS	Mortality; serious infection; malignancy; hypersensitivity including anaphylaxis; IR; depression; immunization	*Systemic*: Nausea; diarrhea; pyrexia; pain in extremity; bronchitis; depression; migraine *Cutaneous*: Rash; pruritus
Benralizumab (Frasenra^®^)	IL-5Rα	Hypersensitivity; helminth infections—treat prior; decrease steroids gradually	*Systemic*: Headache; pharyngitis
Bezlotoxumab (Zinplava^®^)	*Clostridium difficile* toxin B	Heart failure	*Systemic*: Nausea; pyrexia; headache
Bimekizumab (Bimzelx^®^)	IL-17A, IL-17F, IL-17AF	Infections; pre-evaluation for tuberculosis; IBD; avoid live vaccines; hypersensitivity	*Systemic*: Infections and infestations, nervous system disorders; isr *Cutaneous*: Dermatitis; acne; eczema
Brodalumab (Siliq^®^; Kyntheum^®^; Lumicef^®^)	IL-17RA	*Boxed warning*: Suicidal ideation and behavior. *Other*: TB; infections; Crohn’s disease; avoid live vaccines	*Systemic*: Arthralgia; headache; fatigue; diarrhea; oropharyngeal pain; nausea; myalgia; isr, influenza; neutropenia; tinea infections
Brolucizumab-dbll (Beovu^®^)	VEGF-A	Endophthalmitis and retinal detachment; risk of arterial thromboembolic events; increase in intraocular pressure	*Systemic*: Conjunctival hemorrhage; eye pain; vitreous floaters; cataracts; blurred vision
Burosumab-twza (Crysvita^®^)	FGF23	Hypersensitivity; isr; hyperphosphatemia and risk of nephrocalcinosis	*Systemic*: Headache; isr; vomiting; pyrexia; pain in extremity; decreased vitamin D
Canakinumab (Ilaris^®^)	IL-1β	Increased risk of serious infections; immunization; MAS; hypersensitivity; immunosuppression	*Systemic*: CAPS—Nasopharyngitis; diarrhea; influenza; headache; nausea; dizziness/vertigo; SJIA, URTI, isr, abdominal pain
Caplacizumab-yhdp (Cablivi^®^)	von Willibrand factor	Bleeding	*Systemic*: Epistaxis; gingival bleeding; headache; isr
Casirivimab + Imdevimab (REGEN-COV^®^; Ronapreve^®^)	mAbs bind to SARS-CoV-2 spike protein RBD preventing binding to the ACE2 receptor	Hypersensitivity including anaphylaxis; IR	-
Certolizumab pegol (Cimzia^®^)	TNF	*Boxed warning*: Serious infections; lymphoma and other malignancies *Other*: Heart failure; serious allergic reactions; hepatitis B reactivation; demyelinating disease; cytopenias; lupus-like syndrome	*Systemic*: URTI; cardiac disorders; eye disorders; isr; hepatitis and ↑ liver enzymes; nephrotic syndrome; renal failure; thrombophlebitis; vasculitis *Cutaneous*: Dermatitis; erythema nodosum; urticaria
Crizanlizumab-tmca (Adakveo^®^)	P-selectin	IR	*Systemic*: Nausea; arthralgia; back pain; pyrexia
Daclizumab (Zinbryta^®^)	IL-2 receptor α-chain (CD25)	*Boxed warning*: Hepatic injury including autoimmune hepatitis and other immune-mediated disorders *Other*: Hypersensitivity; infections; depression and suicide	*Systemic*: Nasopharyngitis; URTI; oropharyngeal pain; bronchitis; eczema; depression; influenza. *Cutaneous*: Dermatitis, rash
Denosumab (Prolia^®^)	RANKL	Hypersensitivity; hypocalcemia; serious infections; osteonecrosis of jaw; atypical femoral fractures; severe bone, joint, muscle pain; suppression of bone turnover; dermatologic reactions	*Systemic*: Post-menopausal osteoporosis—back, extremity and musculoskeletal pain; hypercholesterolemia; cystitis; male osteoporosis—back pain; arthralgia; nasopharyngitis *Cutaneous*: Rash; pruritus; dermatitis; eczema
Dupilumab (Dupixent^®^)	IL-4Rα	Hypersensitivity; conjunctivitis and keratitis; eosinophilic conditions; helminth infections—treat prior; decrease steroids gradually	*Systemic*: Conjunctivitis; blepharitis; eye pruritus; herpes infections; keratitis; dry eye; oropharyngeal pain; isr; eosinophilia
Eculizumab (Soliris^®^)	Complement C5	*Boxed warning*: Serious meningococcal infection	*Systemic*: PNH—headache; nasopharyngitis; back pain; nausea; AHUS—hypertension; URTI; GI; abdominal pain; anemia; cough; pyrexia; peripheral edema *Cutaneous*: Rash; pruritus
Efgartigimod-alfa-fcab (Vyvgart^®^) ^5^	Neonatal Fc receptor FcRn	See reference below ^5^	See reference below ^5^
Emapalumab-lzsg (Gamifant^®^)	IFNγ	Infections; IR; avoid live vaccines	*Systemic*: Infections; pyrexia; hypertension; IR
Emicizumab-kxwh (Hemlibra^®^)	Factors IXa & X	*Boxed warning*: Thrombotic microangiopathy and thromboembolism. *Other*: mAb interference with coagulation tests	*Systemic*: Arthralgia; isr; headache
Eptinezumab-jjmr (Vyepti^®^)	CGRP	Hypersensitivity	*Systemic*: Nasopharyngitis; hypersensitivity
Erenumab-zooe (Aimoig^®^)	CGRP Receptor	-	*Systemic*: Constipation; isr
Evinacumab-dgnb (Evkeeza^®^)	ANGPTL3	Serious hypersensitivity; embryo-fetal toxicity	*Systemic*: Nasopharyngitis; influenza-like illness; dizziness; rhinorrhea; nausea
Evolocumab (Repatha^®^)	PCSK9	Patients with renal and hepatic impairments have not yet been adequately studied; cover of prefilled syringe and pen contain latex which may cause allergic reactions	*Systemic:* Nasopharyngitis; isr; influenza; URTI; back pain; arthralgia; hypertension; nausea *Cutaneous*: Rash; hives
Fremanezumab-vfrm (Ajovy^®^)	CGRP	Hypersensitivity	*Systemic*: isr
Galcanezumab-gnlm (Emgality^®^)	CGRP	Hypersensitivity	*Systemic*: isr
Golimumab (Simponi^®^)	TNF	*Boxed warning*: Serious infections; lymphoma, and other malignancies *Other*: Invasive fungal infections; heart failure; hepatitis B reactivation; demyelinating disease; hypersensitivity	*Systemic*: URTI; viral infections; bronchitis; ↑ liver enzymes; sarcoidosis; ILD; paresthesia *Cutaneous*: Skin exfoliation; rash
Guselkumab (Tremfya^®^)	IL-23	Infections; prior evaluation for TB	*Systemic*: URTI; isr; arthralgia; headache; diarrhea; tinea; gastroenteritis; herpes simplex infections
Ibalizumab-uiyk (Trogarzo^®^)	CD4	IRIS	*Systemic*: Diarrhea, nausea; dizziness. *Cutaneous*: Rash
Idarucizumab (Praxbind^®^)	Dabigatran	Thromboembolic risk; hypersensitivity; risk of adverse reaction in patients with hereditary fructose intolerance; reappearance of bleeding	*Systemic*: Headache; hypokalemia; delirium; pneumonia; constipation; pyrexia
Inebilizumab-cdon (Uplizna^®^)	CD19	IR; infections; monitor immunoglobulin levels; fetal risk	*Systemic*: Urinary tract infection; arthralgia
Infliximab (Remicade^®^)	TNF	*Boxed warning*: Serious infections; malignancy *Other*: Hepatitis B reactivation; hepatotoxicity; cytopenias; demyelinating disease; lupus-like syndrome	*Systemic*: Infections; pancytopenia; anemia; cellulitis; serum sickness; thrombophlebitis; intestinal obstruction; ILD; anaphylaxis; IRs *Cutaneous*: Cutaneous vasculitis; SJS; EM; psoriasis;
Ixekizumab (Taltz^®^)	IL-17A	Infections: TB—evaluate prior; hypersensitivity; inflammatory bowel disease	*Systemic*: URTI; isr; nausea; tinea infections
Lanadelumab-flyo (Takhzyro^®^)	Plasma kallikrein	Hypersensitivity	*Systemic*: URTI; isr; headache; diarrhea; dizziness; myalgia *Cutaneous*: Rash
Mepolizumab (Nucala^®^)	IL-5	Hypersensitivity; helminth infections—treat prior; herpes zoster infections—consider prior vaccination; decrease steroids gradually; not to be used for bronchospasm or status asthmaticus	*Systemic*: Headache; isr; back pain; fatigue
Natalizumab (Tysabri^®^)	α4 integrin (binds to α4β1 and α4β7 integrins)	*Boxed warning*: PML *Other*: Hypersensitivity; hepatotoxicity; immunosuppression/infections; IRIS	*Systemic*: MS—headache; fatigue; arthralgia; urinary tract infection; URTI; gastroenteritis; vaginitis; diarrhea. CD—headache; URTI; nausea *Cutaneous*: Rash; urticaria
Obiltoxaximab (Anthim^®^)	*Bacillus anthracis* PA	*Boxed warning*: Hypersensitivity and anaphylaxis	*Systemic*: URTI; headache; pruritus; IR pain, swelling, bruise *Cutaneous*: Urticaria
Ocrelizumab (Ocrevus^®^)	CD20	Infections; IR; increased risk of malignancy	*Systemic*: Respiratory tract infections; IR; PML *Cutaneous*: Skin infections
Omalizumab (Xolair^®^)	IgE	Anaphylaxis; malignancy; acute asthma; decrease CSs gradually; eosinophilia; serum sickness-like reaction; parasitic infection	*Systemic*: Allergic asthma— arthralgia; pain; dizziness; fracture; earache. CIU—nausea; pharyngitis; URTI; sinusitis; arthralgia; headache; cough; virus infections *Cutaneous*: Pruritus; dermatitis.
Palivisumab (Synagis^®^)	RSVF	Anaphylaxis; delay administration during moderate–severe infections; give with caution in cases of thrombocytopenia or coagulation disorders	*Systemic*: isr; pyrexia; apnea; cough; dizziness thrombocytopenia *Cutaneous*: Rash; itching; erythema
Ranibizumab (Lucentis^®^)	VEGF-A	Endophthalmitis and retinal detachment, increase in intraocular pressure and risk of arterial thromboembolic events after intravitreal injection	*Systemic*: Conjunctival hemorrhage; eye pain; vitreous floaters; cataracts
Ravulizumab-cwvz (Ultomiris^®^)	Complement C5	*Boxed warning*: Serious meningococcal infections	*Systemic*: URTI; headache; diarrhea; nausea
Raxibacumab (ABthrax^®^)	*Bacillus anthracis* PA	IR	*Systemic*: Pain in extremity; somnolence; headache; URTI; nausea; cough; arthralgias. *Cutaneous*: Rash; pruritus; urticaria
Regdanvirimab (Regkirona^®^)	Binds to SARS-CoV-2 spike protein RBD preventing binding to ACE2 receptor	Hypersensitivity including anaphylaxis; IR	--
Reslizumab (Cinqair^®^)	IL-5	*Boxed warning*: Anaphylaxis *Other*: Helminth infections—treat prior; decrease steroids gradually; malignancy	*Systemic*: Oropharyngeal pain
Risankizumab-rzaa (Skyrizi^®^)	IL-23 p19	Infections; prior evaluation for TB; hypersensensitivity	*Systemic*: URTI; isr; diarrhea
Romosozumab-aqqg (Evenity^®^)	Sclerostin	*Boxed warning*: Potential risk of myocardial infarction, stroke, and cardiovascular death *Other*: Cardiac events; hypersensitivity; hypocalemia; atypical femoral fracture	*Systemic*: Arthralgia; headache
Satralizumab-mwge (Enspryng^®^)	IL-6R	Infections; elevated liver enzymes (ALT, AST); decreased neutrophils	*Systemic*: Nasopharyngitis; headache; URTI; gastritis; arthralgia; extremity pain; fatigue; nausea *Cutaneous*: Rash
Sarilumab (Kevzara^®^)	IL-6R	*Boxed warning*: Risk of serious infection *Other*: GI perforation; avoid live vaccines; hypersensitivity; neutropenia; thrombocytopenia	*Systemic*: increased ALT; isr; URTI; urinary tract infections
Secukinumab (Cosentyx^®^)	IL-17A	Infections; tuberculosis activation; exacerbation of Crohn’s disease; hypersensitivity; avoid live vaccines	*Systemic*: Nasopharyngitis; diarrhea; URTI; rhinitis *Cutaneous*: Urticaria
Sotrovimab (Xevudy^®^) ^6^	Spike protein RBD of SARS-CoV-2	Hypersensitivity reactions including anaphylaxis	--
Teprotumumab-trbw (Tepezza^®^)	IGF-1R	IR; exacerbation of pre-existing inflammatory bowel disease; hyperglycemia	*Systemic*: Muscle spasm; nausea; alopecia; diarrhea; fatigue; hyperglycemia; hearing impairment; dry skin; dysgeusia; headache
Tezepelumab-ekko (Tezspire^®^) ^7^	Thymic stromal lymphopoietin	Hypersensitivity; acute asthma and deteriorating disease; reduction of corticosteroid dosage; parasite infection; live attenuated virus vaccines ^7^	*Systemic*: Pharyngitis; arthralgia; back pain ^7^
Tildrakizumab-asmn (Ilumetri^®^; Ilumya^®^)	IL-23 p19	Infections; prior evaluation for TB; hypersensensitivity	*Systemic*: URTI; isr; diarrhea
Tocilizumab (Actemra^®^; RoActemra^®^)	IL-6R	*Boxed warning*: Serious infections *Other*: GI perforation; avoid live vaccines; hypersensitivity; laboratory monitoring	*Systemic*: Nasophraryngitis; nausea; ↑ liver enzymes; IR; hypertension; thrombocytopenia; neutropenia; headache *Cutaneous*: Dermatologic reactions
Tralokinumab (Adtralza^®^)	IL-13	Hypersensitivity; conjunctivitis; helminth infection; avoid live and live attenuated vaccines	*Systemic*: URTI; conjunctivitis; eosinophilia; isr
Ustekinumab (Stelara^®^)	IL-12 IL-23	Infections; tuberculosis; RPLS; malignancies; anaphylaxis; avoid live vaccines	*Systemic*: Nasopharyngitis; headache; dental infections; URTI; isr; arthralgia; GI *Cutaneous:* Pruritus
Vedolizumab (Entyvio^®^)	α4β7 integrin	Hypersensitivity/IR; infections; PML; liver injury	*Systemic*: Headache; arthralgia; nausea; pyrexia; URTI; cough; bronchitis; influenza; back pain; pain in extremities; nasopharyngitis *Cutaneous*: Rash; pruritus

ACE2—angiotensin-converting enzyme 2; AHUS—atypical hemolytic uremic syndrome; ANGPTL3—angiopoietin-like 3; BLyS—B lymphocyte stimulator, also known as B cell-activating factor, BAFF; C5—complement component 5; CAPS—cryopyrin-associated periodic syndrome; CD—Crohn’s disease; CIU—chronic idiopathic urticaria; COVID—Coronavirus disease; CSs—corticosteroids; CV—cardiovascular; EBOV—*Zaire ebolavirus*; EM—erythema multiforme; GI—gastrointestinal; GP1—glycoprotein 1 of EBOV; HSTC—hematopoietic stem cell transplantation; IBD—inflammatory bowel disease; IFNAR—subunit I type I interferon receptor; IGF-1R—insulin-like growth factor receptor-1; ILD—interstitial lung disease; IR—infusion reaction; IRIS—immune reconstitution inflammatory syndrome; isr—injection site reaction; MAS—macrophage activation syndrome; MS—multiple sclerosis; NS—nervous system; PA—protective antigen of B. anthracis toxin; PCSK9—proprotein convertase subtilisin/kexin type 9; PML—progressive multifocal leukoencephalopathy; PNH—paroxysmal nocturnal hemoglobinuria; RANKL—receptor activator of nuclear factor kappa-B ligand (CD254); RBD—receptor binding domain; REMS—Risk Evaluation Mitigation Strategy; RSVF—human respiratory syncytial virus (F protein coat antigen); SARS-CoV-2—severe acute respiratory syndrome coronavirus 2; SJIA—active systemic juvenile idiopathic arthritis; SJS—Stevens–Johnson syndrome; URTI—upper respiratory tract infection; UTI—urinary tract infection; VEGF-A—vascular endothelial growth factor A. ^1^ Approved by the FDA or EMA or both. ^2^ Monoclonal antibodies are listed in alphabetical order. ^3^ Specificity of antibody. ^4^ Adverse events in addition to those mentioned as occurring, or potentially likely to occur, and shown in column 3. ^5^ Approved by the FDA on 17 December 2021. For safety data and adverse events, see Howard, J.F; Bril, V.; Vu, T.; et al. [21]. ^6^ Note added in press: Approved by the EMA on 17 December 2021. ^7^ Approved by the FDA on 17 December 2021. For safety data and adverse events, see Menzies-Gow, A.; Colice G, Griffiths, J.M.; et al. [22] and Menzies-Gow, A.; Corren, J.; Bourdin, A.; et al. [23]. ↑ increase.

**Table 5:** Adverse events associated with approved ^1^ monoclonal antibodies used for cancer therapy (as at December 2021).

Monoclonal Antibody ^2^ INN and Trade Names	Target ^3^	Warnings, Precautions, Risks, and Safety Concerns	Other Adverse Events ^4^: Serious and Common
Ado-trastuzumab emtansine (Kadcyla^®^)	HER2	*Boxed warning:* Hepatotoxicity; cardiac toxicity; embryo-fetal toxicity *Other:* IR; pulmonary toxicity; extravasation; hemorrhage; thrombocytopenia; neurotoxicity	*Systemic*: Pulmonary events; fetal harm; LVD; hypersensitivity/IR; nausea; fatigue; anemia; headache; musculoskeletal pain; increased transaminases; constipation *Cutaneous*: Rash; pruritus
Alemtuzumab (Campath^®^; MabCampath^®^)	CD52	*Boxed warning*: Cytopenias; IR; immunosuppression/infections *Other:* Immunization	*Systemic*: Pulmonary events; immunogenicity; cardiac events; diarrhea; nausea; emesis; insomnia *Cutaneous*: Rash; urticaria; erythema; pruritus
Amivantamab-vmjw (Rybrevant^®^)	EGFR and c-MET receptors	ILD/pneumonitis; IR; dermatologic (including acneiform dermatitis and TEN); ocular toxicity; embryo-fetal toxicity	*Systemic*: IR; paronychia; musculoskeletal pain; dyspnea; nausea; fatigue; edema; stomatitis; cough; constipation; vomiting *Cutaneous*: Rash
Atezolizumab (Tecentriq^®^)	PD-L1	Immune-mediated pneumonitis, colitis, hepatitis, endocrinopathies (hypophysitis, thyroid disorders, adrenal insufficiency, diabetes mellitus); embryo-fetal toxicity	*Systemic*: IR; fatigue; nausea; infections; urinary tract infections; decreased appetite; diarrhea; pyrexia; constipation; dyspnea. *Cutaneous*: Rash; pruritus
Avelumab (Bavencio^®^)	PD-L1	Immune-mediated pneumonitis, colitis, hepatitis, endocrinopathies, nephritis and renal dysfunction; IR	*Systemic*: Fatigue; musculoskeletal pain; diarrhea; nausea; decreased appetite; peripheral edema; urinary tract infection *Cutaneous*: Rash; pruritus
Belantamab mafodoton-blmf (Blenrep^®^)	BCMA with MMAF microtubule inhibitor	*Boxed warning*: Ocular toxicity *Other:* Thrombocytopenia; IR; embryo-fetal toxicity	*Systemic*: Keratopathy; decreased visual acuity, nausea; blurred vision; pyrexia; IR; fatigue; decreased platelets, lymphocytes, hemoglobin; increased creatinine, GGT
Bevacizumab (Avastin^®^)	VEGF-A	*Boxed warning:* GI perforation; surgery/wound healing; hemorrhage *Other*: Non-GI fistula; RPLS; IR; CHF; hypertension; arterial/venous thromboembolism; eye disorders; proteinurea; neutropenia/infections; ONJ	*Systemic*: Pulmonary events; epistaxis; headache; rectal hemorrhage; dry skin; necrotizing fasciitis; taste alteration; lacrimation disorder; ovarian failure *Cutaneous*: Exfoliative dermatitis; alopecia
Blinatumomab (Blincyto^®^)	CD19/CD3 epsilon	*Boxed warning:* CRS; neurological toxicities *Other:* Infections; neutropenia and febrile neutropenia; TLS; elevated liver enzymes; leukoencephalopathy	*Systemic*: HLH; pyrexia; lymphopenia; leukopenia; chills; headache; CNS symptoms (disorientation, confusion, tremor, speech disorders); hypokalemia; pneumonia; sepsis, constipation, peripheral edema *Cutaneous*: Rash
Brentuximab vedotin(Adcetris^®^)	CD-30	*Boxed warning:* PML *Other:* Peripheral neuropathy; IR and anaphylaxis; neutropenia; infections; fetal harm; hepatotoxicity; TLS; SJS	*Systemic*: Cytopenias; immunogenicity; URTI; pyrexia; nausea; vomiting; fatigue; cough; anaphylaxis *Cutaneous*: Rash; pruritus; SJS; alopecia
Catumaxomab (Removab^®^)	EpCAM/CD3	Monitor and evaluate for: CRS; SIRS; HAMA/HARA; GI hemorrhage; hepatic disorders; abdominal infection; ileus/intestinal perforation; decreased lymphocyte count	*Systemic*: Cytopenias; hepatotoxicity; abdominal disorders; pyrexia; chills; nausea; vomiting; infections; immunogenicity; dyspnea *Cutaneous*: Rash; erythema; allergic dermatitis; hyperhidrosis; pruritus
Cemiplimab-rwlc (Libtayo^®^)	PD-1	Immune-mediated pneumonitis, colitis, hepatitis, endocrinopathies, nephritis, dermatologic reactions; IR; embryo-fetal toxicity	*Systemic*: Diarrhea; fatigue; nausea; constipation; musculoskeletal pain *Cutaneous*: Rash; pruritus
Cetuximab (Erbitux^®^)	EFGR	*Boxed warning:* Serious IR and cardiopulmonary arrest. *Other:* Pulmonary toxicity; dermatologic toxicity; hypomagnesemia	*Systemic*: Electrolyte imbalance; infection; GI; anaphylaxis; headache; diarrhea *Cutaneous*: Acneiform rash; nail changes; xeroderma; paronychial inflammation; pruritus
Daratumumab (Darzalex^®^)	CD38	IR; interference with serological testing; neutropenia; thrombocytopenia	*Systemic*: Neutropenia; thrombocytopenia; fatigue; nausea; diarrhea; constipation; vomiting; muscle spasms; arthralgia; back pain; pyrexia; chills; dizziness; insomnia; cough; dyspnea; peripheral edema; peripheral sensory neuropathy; URTI
Denosumab (Prolia^®^; Xgeva^®^)	RANKL	Hypocalcemia; ONJ; embryo-fetal toxicity	*Systemic*: Osteomyelitis; hypophosphatemia; dyspnea; fatigue/asthenia; back pain; nausea; extremity pain *Cutaneous*: Rash; pruritus; dermatitis; eczema
Dinutuximab (Unituxin^®^)	GD2	*Boxed warning:* Serious IR; neuropathy *Other:* CLS and hypotension; infection; RPLS; neurological disorders of eye; BMS; electrolyte abnormalities; AHUS; embryo-fetal toxicity	*Systemic*: Hypokalemia; pain; fever; hypocalcemia; hyponatremia; anemia; thrombocytopenia; lymphopenia; neutropenia; increased AST, ALT; GI *Cutaneous*: Urticaria
Dostarlimab-gxly (Jemperli^®^)	PD-1	Immune-mediated colitis, pneumonitis, hepatitis, endocrinopathies, nephritis, dermatologic adverse reactions; IR; complications of allogeneic HSCT after PD-1/L-1–blocking antibody; embryo-fetal toxicity	*Systemic*: Fatigue/asthenia; nausea; diarrhea; anemia
Durvalumab (Imfinzi^®^)	PD-L1	Immune-mediated pneumonitis, colitis, hepatitis, nephritis, endocrinopathies; dermatologic reactions; embryo-fetal toxicity; infections; IR	*Systemic*: Fatigue; musculoskeletal pain; diarrhea; nausea; decreased appetite; peripheral edema; urinary tract infection; pneumonitis; dyspnea; URTI; cough *Cutaneous*: Rash; pruritus
Elotuzumab (Empliciti^®^)	SLAMF7	IR; infections; second primary malignancies; hepatotoxicity; interference in monitoring M-protein impacting determination of complete response in patients with IgGκ myeloma protein	*Systemic*: Fatigue; diarrhea; pyrexia; constipation; cough; peripheral neuropathy; nasopharyngitis; URTI; decreased appetite; pneumonia
Enfortumab-vedotin-ejfv (Padcev^®^)	Nectin-4 with MMAE microtubule inhibitor	Hyperglycemia; peripheral neuropathy; ocular disorders; skin reactions; infusion site extravasation; embryo-fetal toxicity	*Systemic*: Fatigue; peripheral neuropathy; decreased appetite; rash; alopecia; nausea; dysgeusia; diarrhea; dry eye *Cutaneous*: Pruritus; dry skin
Fam-trastuzumab deruxtecan-nxki (Enhertu^®^)	HER2	*Boxed warning:* ILD and pneumonitis; embryo-fetal toxicity *Other*: Neutropenia; LVD	*Systemic*: Decreased hemoglobin, white blood cells, neutrophils, lymphocytes, platelets; nausea; vomiting; constipation; fatigue; decreased appetite; anemia; diarrhea; hypokalemia; pyrexia; alopecia; increased blood bilirubin, aspartate aminotransferase, AP, alanine aminotransferase
Gemtuzumab ozogamicin (Mylotarg^®^)	CD33	*Boxed warning:* Hepatotoxicity including severe or fatal hepatic veno-occlusive disease *Other:* IR including anaphylaxis; hemorrhage; embryo-fetal toxicity	*Systemic*: Hemorrhage; infection; fever; nausea; vomiting; constipation; headache; increased ALT, AST; mucositis *Cutaneous*: Rash
Ibritumomab tiuxetan (Zevalin^®^)	CD20	*Boxed warning:* Serious IR; severe cytopenias; severe mucocutaneous and cutaneous reactions *Other:* MDS and AML; extravasation; immunization	*Systemic*: Infections; asthenia; musculoskeletal symptoms; GI; hemorrhage; hypersensitivity *Cutaneous*: Exfoliative dermatitis; bullous dermatitis; EM; SJS; TEN
Inotuzumab ozogamicin (Besponsa^®^)	CD22	*Boxed warning:* Hepatotoxicity including hepatic veno-occlusive disease; increased risk of post- transplant non-relapse mortality *Other:* myelosuppression; embryo- fetal toxicity; QT interval prolongation	*Systemic*: IR; cytopenias; nausea; fatigue; hemorrhage; pyrexia; infection; headache; febrile neutropenia; increased transaminases; hyperbilirubinemia
Ipilimumab (Yervoy^®^)	CTLA-4	*Boxed warning*: Immune-mediated adverse reactions	*Systemic*: Diarrhea; fatigue; colitis *Cutaneous*: Rash; pruritus; dermatitis
Isatuximab-irfc (Sarclisa^®^)	CD38	IR; neutropenia; second primary malignancies; indirect antiglobulin test and interference with serum electrophoresis and immunofixation tests	*Systemic*: Neutropenia; IR; pneumonia; URTI; diarrhea; anemia; lymphopenia; thrombocytopenia
Loncastumab tesirine-lpyl (Zynlonta^®^)	CD19 with teserine cytotoxic agent	Effusions (pericardial, pleural, ascites); embryo-fetal toxicity; myelosuppression; infections; cutaneous reactions (including photosensitivity)	*Systemic*: Thrombocytopenia; increased gamma-glutamyltransferase; neutropenia; nausea; anemia; hyperglycemia; transaminase elevation; fatigue; hypoalbuminemia; edema; musculoskeletal pain *Cutaneous*: Rash
Margetuximab-cmkb (Margenza^®^)	HER2	*Boxed warning*: LVD; embryo-fetal toxicity	*Systemic*: Fatigue/asthenia; nausea; diarrhea; vomiting; constipation; IR; headache; pyrexia; alopecia; abdominal pain; peripheral neuropathy; arthralgia/myalgia; cough; decreased appetite; dyspnea; extremity pain *Cutaneous*: PPE
Mogamulizumab-kpkc (Poteligeo^®^)	CCR4	Dermatologic toxicity; IR; infections; autoimmune reactions; HSCT complications	*Systemic*: IR; diarrhea; fatigue; URTI; musculoskeletal pain *Cutaneous*: Rash
Moxetumomab pasudox- tdfk (Lumoxiti^®^)	CD22	*Boxed warning:* CLS; hemolytic uremic syndrome *Other:* Renal toxicity; electrolyte abnormalities; IR	*Systemic*: Edema; nausea; fatigue; headache; pyrexia; constipation; diarrhea; anemia; increased creatinine, ALT, AST; hypophosphatemia; hypocalcemia
Naxitamab-gqgk (Danyelza^®^)	GD2	*Boxed warning:* Serious IR; neurotoxicity including RPLS	*Systemic*: IR; isr; pain; tachycardia; vomiting; cough; nausea; diarrhea; decreased appetite; hypertension; fatigue; peripheral neuropathy; edema; urticaria; pyrexia; headache; anxiety; irritability; decreased lymphocytes; neutrophils, hemoglobin, platelets, K, Ca, Na, glucose, albumin, phosphate; increased alanine aminotransferase *Cutaneous*: EM
Necitumumab (Portrazza^®^)	EGFR	*Boxed warning*: Cardiopulmonary arrest; hypo-magnesemia *Other*: Venous, arterial thromboembolic events; dermatologic toxicities; embryo-fetal toxicity; ↑ toxicity, mortality in patients with non- squamous NSCLC; IR	*Systemic*: Diarrhea; vomiting *Cutaneous*: Rash; dermatitis acneiform
Nivolumab (OPDIVO^®^)	PD-1	Immune-mediated adverse reactions; embryo-fetal toxicity	*Systemic*: Increased ALT, AST, AP; hyponatremia; hyper- and hypokalemia; hyper- and hypocalcemia; lymphopenia; fatigue; asthenia; musculoskeletal and abdominal pain; dyspnea; cough; GI. *Cutaneous*: Rash; pruritus
Obinutuzumab (Gazyva^®^; Gazyvaro^®^)	CD20	*Boxed warning:* Hepatitis B virus reactivation; PML. *Other:* IR; TLS; neutropenia; thrombocytopeia; infections; immunization	*Systemic*: Anemia; pyrexia; musculoskeletal disorders; headache; cough
Ofatumumab (Arzerra^®^)	CD20	IR; Hepatitis B virus reactivation; PML; cytopenias intestinal obstruction; immunization	*Systemic*: Infections; pneumonia; neutropenia; pyrexia; dyspnea; cough; diarrhea; URTI; nausea; fatigue; bronchitis *Cutaneous*: Rash; urticaria; hyperhidrosis.
Olaratumab (Lartruvo^®^)	PDGFR-α	IR; embryo-fetal toxicity	*Systemic*: Olaratumab + doxorubicin: fatigue; musculoskeletal pain; diarrhea; decreased appetite; headache; neuropathy; cytopenias; hyperglycemia; elevated aPTT; hypokalemia; hypophosphatemia *Cutaneous*: Alopecia
Panitumumab (Vectibix^®^)	EGFR	*Boxed warning:* Dermatologic toxicity; IR *Other:* Increased toxicity with bevacizumab and chemotherapy; pulmonary toxicities; electrolyte depletion; ocular events	*Systemic*: Pulmonary events; pulmonary embolism; GI; fatigue; abdominal pain; hypomagnesemia *Cutaneous*: Rash; dermatitis ‘acneiform’; erythema; exfoliation; paronychia; skin fissures; photosensitivity; xerosis; pruritus
Pembrolizumab (Keytruda^®^)	PD-1	Immune-mediated adverse reactions; embryo-fetal toxicity	*Systemic*: Fatigue; peripheral edema; chills; pyrexia; renal failure; cellulitis; decreased appetite; dyspnea; arthralgia; nausea; diarrhea; cough *Cutaneous*: Rash; pruritus; vitiligo
Pertuzumab (Perjeta^®^)	HER2	*Boxed warning*: Cardiomyopathy; embryo-fetal toxicity. *Other:* IR; hypersensitivity/anaphylaxis	*Systemic*: Neutropenias; LVD; peripheral neuropathy; fatigue; GI; asthenia *Cutaneous*: Rash; paronychia; pruritus; alopecia; PPE (in combination therapy)
Polatuzumab vedotin-piiq (Polivy^®^)	CD79b	Peripheral neuropathy; myelosuppression and related reactions; infections; IR; TLS; PML; hepatotoxicity; embryo-fetal toxicity	*Systemic*: Cytopenia; fatigue; decreased appetite; diarrhea; pyrexia; pneumonia
Ramucirumab (Cyramza^®^)	VEGFR2	*Boxed warning:* Hemorrhage; GI perforation; impaired wound healing. *Other:* A rterial thromboembolic events; IR; RPLS; hypertension; deterioration in patients with cirrhosis; proteinuria including nephrotic syndrome; thyroid dysfunction; embryo-fetal risk	*Systemic:* Hypertension; diarrhea; headache; hytremia; neutropenia; epistaxis; stomatitis; immunogenicity
Rituximab (Rituxan^®^; MabThera^®^)	CD20	*Boxed warning:* Fatal IRs; TLS; potentially fatal PML and severe mucocutaneous reactions *Other:* hepatitis B virus reactivation; infections; cardiac arrhythmias; bowel obstruction and perforation	*Systemic*: Pulmonary events; renal toxicity; neutropenias; serum sickness; anaphylaxis; fever; lymphopenia; chills; asthenia *Cutaneous*: Paraneoplastic pemphigus; lichenoid dermatitis; vesicullobullous dermatitis; SJS; TEN
Sacituzumab govitecan-hziy (Trodelvy^®^)	Trop-2 with topoisomerase inhibitor	*Boxed warning:* Severe neutropenia; severe diarrhea *Other:* Hypersensitivity; nausea/vomiting; risk of neutropenia increased in individuals with reduced uridine diphosphate-glucuronosyl transferase 1A1; embryo-fetal toxicity	*Systemic*: Nausea; neutropenia; diarrhea; fatigue; anemia; vomiting; alopecia; constipation; decreased appetite; abdominal pain *Cutaneous*: Rash
Siltuximab (Sylvant^®^)	IL-6	Not for patients with severe infections or live vaccines; IR; cautionary use in patients with GI perforation risk	*Systemic*: Hyperuricemia; URTI; increased weight *Cutaneous*: Rash; pruritus
Tafasitamab-cxix (Monjuvi^®^)	D19	IR; myelosuppression; infections; embryo-fetal toxicity	*Systemic*: Neutropenia; fatigue; anemia; diarrhea; thrombocytopenia; cough; pyrexia; peripheral edema; URTI; decreased appetite
Tisotumab vedotin-tftv (Tivdak^®^)	TF with MMAE microtubule inhibitor	*Boxed warning:* Ocular toxicity *Other*: Ocular adverse reactions, e.g., conjunctival reactions, dry eyes, corneal reactions, blepharitis; ulcerative keratitis; peripheral neuropathy; pneumonitis; embryo- fetal toxicity	*Systemic*: Most serious: ileus; hemorrhage; pneumonia; sepsis; pyrexia; peripheral neuropathy; constipation. Most common: diarrhea; peripheral neuropathy; conjunctival and corneal reactions; fatigue; alopecia; epistaxis; decreased hemoglobin, lymphocytes, and leukocytes; increased creatinine; dry eye; prothrombin international normalized ratio; *aPTT* prolonged *Cutaneous*: Rash
Trastuzumab (Herceptin^®^)	HER2	*Boxed warning:* Cardiomyopathy; IR; pulmonary toxicity *Other:* Exacerbation of chemotherapy-induced neutropenia; embryo-fetal toxicity	*Systemic*: Neutropenia; anemia; thrombocytopenia; pulmonary events; LVD; GI; chills; fever; URTI; anaphylaxis/angioedema; headache; cough; stomatitis; mucosal inflammation *Cutaneous*: Rash; nail disorders; pruritus

AHUS—atypical hemolytic syndrome; ALT—alanine transaminase; AML—acute myelogenous leukemia; AP—alkaline phosphatase; aPTT—activated partial thromboplastin time; AST—aspartate transaminase; BCMA—B cell maturation antigen; BMS—bone marrow suppression; CHF—congestive heart failure; CLS—capillary leak syndrome; c-MET—mesenchymal-epithelial transition factor, a tyrosine kinase receptor; CNS—central nervous system; CRS—cytokine release syndrome; CTLA-4—cytotoxic T lymphocyte-associated antigen 4; DLBCL—diffuse large B cell lymphoma; EGFR—epidermal growth factor receptor (HER1, ErbB-1); EM—erythema multiforme; EpCAM—epithelial cell adhesion molecule; GD2—disialoganglioside expressed on tumors of neuroectodermal origin; GGT—gamma-glutamyl transferase; GI—gastrointestine/gastrointestinal symptoms, e.g., nausea, diarrhea, vomiting, constipation; GM-CSF—granulocyte-macrophage colony-stimulating factor; HAMA—human antimouse antibody; HARA—human antirat antibody; HER2—human epidermal growth factor 2, also known as Neu, ErbB2, CD340, or p185; HLH—hemophagocytic lymphohistiocytosis; IR—infusion reactions; isr—injection site reaction; ILD—interstitial lung disease; LBCL—large B cell lymphoma; LVD—left ventricular dysfunction; MMAE—monomethyl auristatin E; MMAF—monomethyl auristatin F; MDS—myelodysplastic syndrome; mTNBC—metastatic triple-negative breast cancer; ONJ—osteonecrosis of the jaw; PD-1—programmed cell death protein 1; PD-L1; PDGFRA—platelet-derived growth factor receptor A; PML—progressive multifocal leucoencephalopathy; PPE—palmar plantar erythrodysaesthesia; RANKL—receptor activator of nuclear factor kappa-B ligand (CD254); RPLS—reversible posterior leukoencephalopathy syndrome; SIRS—systemic inflammatory response syndrome; SJS—Stevens–Johnson syndrome; TEN—toxic epidermal necrolysis; teserine—also known as SG3249, a pyrrolobenzodiazepine dimer; TF—tissue factor, platelet tissue factor, factor III, CD142; TLS—tumor lysis syndrome; Trop-2—trophoblast cell surface antigen-2; URTI—upper respiratory tract infection; VEGF—vascular endothelial growth factor; VEGFR-2—vascular endothelial growth factor receptor 2. ^1^ Approved by the FDA or EMA or both. ^2^ Monoclonal antibodies are listed in alphabetical order. ^3^ Specificity of antibody. ^4^ Adverse events in addition to those mentioned as warnings and precautions in column 3. ↑ increase.

**Table 6:** Individual approved monoclonal antibodies associated with adverse events affecting different organs and tissues.

Anaphylaxis ^1^	Infusion Reactions	Cytopenias	Pulmonary Adverse Events ^2^	Cardiac Adverse Events	Hepatotoxicity ^2^	Other Immune- Mediated Reactions ^3^	Embryo-Fetal Toxicity	Dermatologic Toxicity ^3^
*Monoclonal* *Antibodies for* *non-cancer* *therapy*								
Adalimumab Belimumab Casirivimab+ Imdevimab ^4^ Certolizumab pegol Evinacumab-dgnb Infliximab Obiltoxaximab Omalizumab Palivisumab Regdanvirimab Reslizumab Tocilizumab Ustekinumab	Alemtuzumab ^5^ Anifrolumab-fnia Ansuvimab-zykl Atoltivimab ^6^ Belimumab Casirivimab+ Imdevimab ^4^ Crizanlizumab-tmca Emapalumab-lzsg Inebilizumab-cdon Infliximab Obiltoxaximab Ocrelizumab Raxibacumab Regdanvirimab Sotrovimab Teprotumumab-trbw Tocilizumab Vedolizumab	Abciximab Adalimumab Alemtuzumab ^5^ Brodalumab Certolizumab pegol Infliximab Palivisumab Sarilumab Satralizumab-mwge Tocilizumab	Adalimumab Alemtuzumab ^5^ Golimumab Infliximab	Adalimumab Bezlotoxumab Certolizumab pegol Golimumab Romosozumab-aqqg	Adalimumab Certolizumab pegol Daclizumab Evolocumab Infliximab Natalizumab Vedolizumab	Adalimumab Alemtuzumab Alirocumab Daclizumab Infliximab Omalizumab	Evinacumab-dgnb Inebilizumab-cdon	Abciximab Adalimumab Alemtuzumab ^5^ Alirocumab Bimekizumab Certolizumab pegol Daclizumab Denosumab ^7^ Evolocumab Golimumab Infliximab Natalizumab Obiltoxaximab Ocrelizumab Omalizumab Raxibacumab Secukinumab Tocilizumab
*Monoclonal* *Antibodies for* *cancer therapy*								
Brentuximab vedotin Cetuximab Gemtuzumab ozogamicin Pertuzumab Rituximab Trastuzumab	Ado-trastuzumab Alemtuzumab ^8^ Amivantamab-vmjw Atezolizumab Avelumab Belantamab mafodoton-blmf Bevacizumab Brentuximab vedotin Cemiplimab-rwlc Cetuximab Daratumumab Dinutuximab Dostarlimab-gxly Durvalumab Elotuzumab Gemtuzumab ozogamicin Ibritumomab tiuxetan Inotuzumab ozogamicin Isatuximab-irfc Mogamulizumab-kpkc Moxetumomab pasudox-tdfk Naxitamab-gqgk Necitumumab Obinutuzumab Ofatumumab Olaratumab Panitumumab Pertuzumab Polatuzumab vedotin-piiq Ramucirumab Rituximab Siltuximab Tafasitamab-cxix Trastuzumab	Ado-trastuzumab Alemtuzumab ^8^ Belantamab mafodoton-blmf Blinatumomab Brentuximab vedotin Catumaxomab Daratumumab Dinutuximab Fam-trastuzumab deruxtecan-nxki Ibritumomab tiuxetan Inotuzumab ozogamicin Isatuximab-irfc Loncastumab-tesirine-lpyl Naxitamab-gqgk Obinutuzumab Ofatumumab Olaratumab Pertuzumab Polatuzumab vedotin-piiq Ramucirumab Rituximab Sacituzumab govetican-hziy Tafasitamab-cxix Tisotumab vedotin-tftv Trastuzumab	Ado-trastuzumab Alemtuzumab ^8^ Amivantamab-vmjw Atezolizumab Avelumab Bevacizumab Cemiplimab-rwlc Cetuximab Dostarlimab-gxly Durvalumab Fam-trastuzumab deruxtecan-nxki Ipilimumab Nivolumab Panitumumab Pembrolizumab Rituximab Tisotumab vedotin-tftv Trastuzumab	Ado-trastuzumab Alemtuzumab ^8^ Bevacizumab Cetuximab Fam-trastuzumab deruxtecan-nxki Ibritumomab tiuxetan Inotuzumab ozogamicin Margetuximab-cmkb Necitumumab Obinutuzumab Pertuzumab Ramucirumab Rituximab Romosozumab-aqqg Trastuzumab	Ado-trastuzumab Atezolizumab Avelumab Brentuximab vedotin Catumaxomab Cemiplimab-rwlc Dostarlimab-gxly Durvalumab Elotuzumab Gemtuzumab ozogamicin Inotuzumab Ozogamicin Obinutuzumab Ofatumumab Polatuzumab vedotin-piiq Rituximab	Atezolizumab Avelumab Cemiplimab-rwlc Dostarlimab-gxly Durvalumab Ipilimumab Mogamulizumab-kpkc Nivolumab Pembrolizumab	Amivantamab-vmjw Atezolizumab Belantamab mafodoton-blmf Cemiplimab-rwlc Denosumab ^9^ Dinutuximab Dostarlimab-gxly Durvalumab Enfortumab vedotin-ejfv Fam-trastuzumab Gemtuzumab ozogamicin Inotuzumab ozogamicin Loncastumab tesirine-lpyl Margetuximab-cmkb Necitumumab Nivolumab Olaratumab Pembrolizumab Pertuzumab Polatuzumab vedotin-piiq Ramucirumab Sacituzumab govetican-hziy Tafasitamab-cxix Tisotumab vedotin-tftv Trastuzumab	Alemtuzumab ^8^ Amivantamab-vmjw Bevacizumab Brentuximab vedotin Catumaxomab Cemiplimab-rwlc Cetuximab Denosumab ^9^ Dostarlimab-gxly Durvalumab Enfortumab vedotin-ejfv Ibritumomab tiuxetan Ipilimumab Loncastumabtesirine-lpyl Margetuximab-cmkb Mogamulizumab-kpkc Naxitamab-gqgk Necitumumab Panitumumab Pembrolizumab Pertuzumab Rituximab Trastuzumab

For infusion reactions, cytopenias, pulmonary events, and dermatologic toxicity, alemtuzumab as Lemtrada^®^ and Campath^®^ are counted as one mAb not two; likewise, denosumab as Prolia^®^ and Xgeva^®^ are counted as one mAb in inducing dermatologic toxicity. ^1^ A type I immediate hypersensitivity. ^2^ Includes some mAb-induced hypersensitivities. ^3^ mAbs including, and in addition to, those clearly identified as inducing an adverse event via a type I, II, III, or IV hypersensitivity mechanism. ^4^ A combination of two mAbs directed to the spike protein receptor binding domain of SARS-CoV-2. ^5^ As Lemtrada^®^. ^6^ A combination of Zaire ebolavirus glycoprotein-1-directed human monoclonal antibodies (atoltivimab, maftivimab, and odesivimab), indicated for the treatment of infection caused by Zaire ebolavirus. ^7^ As Prolia^®^. ^8^ As Campath^®^. ^9^ As Xgeva^®^.

**Table 7:** Approved monoclonal antibodies subject to FDA warnings and precautions for cytopenias.

Cytopenia	Thrombocytopenia	Neutropenia	Lymphocytopenia
Adalimumab	Abciximab	Bevacizumab	Catumaxomab
Alemtuzumab ^1^	Ado-trastazumab emtansine	Blinatumomab	
Certolizumab pegol	Belantamab mafodoton-blmf	Brentuximab vedotin	
Ibritumomab tiuxetan ^1^	Daratumumab	Daratumumab	
Infliximab	Palivisumab	Fam-trastuzumab Deruxtecan-nxki	
Ofatumumab	Sarilumab	Isatuximab-irfc	
		Obinutuzumab	
		Sacituzumab govitecan-hziy ^1^	
		Sarilumab	
		Satralizumab-mwge	
		Trastuzumab	

^1^ Subject to boxed warning.

**Table 8:** Cardiac adverse events caused by approved monoclonal antibodies used for therapy.

Monoclonal Antibody	Cardiac Adverse Events
Adalimumab.	Heart failure
Ado-trastuzumab emtansine ^1^	Decreased LVEF
Alemtuzumab	Cardiomyopathy, decreased LVEF, cardiac arrhythmias associated with infusions
Bevacizumab	CHF: incidence of grade 3 reaction for LVD 1%
Bezlotoxumab	Heart failure
Brentuximab vedotin	Supraventricular arrhythmia in systemic anaplastic large cell lymphoma
Certolizumab pegol	Heart failure
Cetuximab	Cardiopulmonary arrest/sudden death
Fam-trastuzumab deruxtecan-nxki	LVD
Golimumab	Heart failure
Ibritumomab tiuxetan	Cardiac arrest related to infusions
Inotuzumab ozogamicin	QT interval prolongation
Margetuximab-cmkb ^1^	LVD
Necitumumab ^1^	Cardiopulmonary arrest
Obinutuzumab	Worsening of preexisting cardiac conditions leading to fatal cardiac events
Pertuzumab ^1^	Cardiomyopathy manifesting as CHF and decreased LVEF
Ramucirumab	Serious, sometimes fatal, myocardial infarction
Rituximab	Cardiac arrhythmias and angina, fatal cardiac failure
Romosozumab-aqqg ^1^	Myocardial infarction, cardiac events, cardiovascular death
Trastuzumab ^1^	Cardiomyopathy manifesting as CHF and decreased LVEF

CHF—congestive heart failure; LVD—left ventricular dysfunction; LVEF—left ventricular ejection fraction. ^1^ FDA boxed warnings apply.

**Table 9:** Liver adverse events induced by approved monoclonal antibodies used for therapy.

Monoclonal Antibody	Liver Adverse Events
Adalimumab	Reactivates hepatitis B; liver failure
Ado-trastuzumab	Hepatotoxicity
Atezolizumab	Immune-mediated hepatitis
Avelumab	Immune-mediated hepatitis
Brentuximab vedotin	Hepatotoxicity
Catumaxomab	Hepatic disorders—hepatotoxicity
Cemiplimab-rwlc	Immune-mediated hepatitis
Certolizumab pegol	Reactivates hepatitis B
Daclizumab	Hepatic injury including autoimmune hepatitis
Dostarlimab-gxly	Immune-mediated hepatitis
Durvalumab	Immune-mediated hepatitis
Elotuzumab	Hepatotoxicity
Evolocumab	Hepatic impairment
Gemtuzumab ozogamicin	Hepatotoxicity including severe or fatal hepatic veno-occlusive disease
Golimumab	Reactivates hepatitis B
Infliximab	Hepatotoxicity
Inotuzumab ozogamicin	Hepatotoxicity including severe or fatal hepatic veno-occlusive disease
Natalizumab	Hepatotoxicity
Obinutuzumab	Reactivates hepatitis B
Ofatumumab	Reactivates hepatitis B
Polatuzumab vedotin-piiq	Hepatotoxicity
Rituximab	Reactivates hepatitis B
Vedolizumab	Possibility of liver injury suggested by elevated levels of transaminase and/or bilirubin

## Data Availability

Any relevant data is available from author on request.
